# Filling the Silence: Reactivation, not Reconstruction

**DOI:** 10.3389/fpsyg.2016.00027

**Published:** 2016-01-26

**Authors:** Dario L. J. F. Paape

**Affiliations:** Department of Linguistics, University of PotsdamPotsdam, Germany

**Keywords:** ellipsis processing, garden-path effect, German, retrieval, reconstruction, self-paced reading

## Abstract

In a self-paced reading experiment, we investigated the processing of sluicing constructions (“sluices”) whose antecedent contained a known garden-path structure in German. Results showed decreased processing times for sluices with garden-path antecedents as well as a disadvantage for antecedents with non-canonical word order downstream from the ellipsis site. A *post-hoc* analysis showed the garden-path advantage also to be present in the region right before the ellipsis site. While no existing account of ellipsis processing explicitly predicted the results, we argue that they are best captured by combining a local antecedent mismatch effect with memory trace reactivation through reanalysis.

## 1. Introduction

Besides verb-phrase ellipsis, sluicing (Ross, 1969) is probably the most-studied ellipsis variety in both theoretical linguistics (e.g., Chung et al., 1995; Merchant, 2001; Potsdam, 2007) and psycholinguistics (e.g., Poirier et al., 2010; Dickey and Bunger, 2011; Yoshida et al., 2013). In sluicing, an entire clause is left out and a wh-element remains behind, as in (1).

(1) John saw Mary, but I don't remember when ____.
       ____ = John saw Mary

Sluicing is anaphoric: to interpret (1), the semantics of the antecedent (*John saw Mary*) must somehow be inserted into the gap behind the word *when* to derive the meaning *I don't remember when John saw Mary*. We write “meaning” because deriving an interpretation is the fundamental goal of sentence processing, not because it is necessarily clear that the relevant representation of the antecedent is semantic in nature. There is an ongoing debate as to whether syntactic structure is also present at ellipsis sites (cf. Cai et al., 2013, and references therein), or whether one should adopt a more discourse-centered approach to the gap-filling process (e.g., Hardt, 1993; Kehler, 2000). Since the evidence to date, at least in our view, does not unequivocally favor any of these views, we will not take a stance with regard to the representation question. We will, however, use syntactic terminology throughout the article for ease of reference.

Even with the question of *what* is inserted into the gap set aside, another point of debate has been *how* it ends up there. Ross (1967) was perhaps the first to explicitly propose a deletion approach to ellipsis (in this case, verb-phrase ellipsis): the missing bit of structure is assumed to be underlyingly present, but its phonological representation is erased under identity with the antecedent^1^. Under the approach taken by Williams (1977), ellipsis involves copying. Like Ross (1967), Williams assumes invisible syntax at the gap, but the terminal symbols of this structure are null elements (Wasow, 1972). The ellipsis is interpreted by copying the terminals (that is, words) from the antecedent to the appropriate positions within the gap.

From a processing perspective, it is not enough to claim that the syntax is there in the silence: the processor must have some way of creating it. A reader of (1) would have to first infer that deletion has applied, then identify the antecedent and finally reconstruct it at the gap. The main aim of the current study is to investigate how this “reconstruction” is to be conceived of: does the parser rebuild the antecedent's structure at the ellipsis site, or does it come to be there by virtue of some other mechanism?

One might think of dispensing with the idea of invisible structure altogether. The approach of Hardt (1993) is explicitly non-syntactic in nature and treats ellipsis as an unstructured proform that refers to a stored meaning in a discourse model. The notion of copying does not enter into the picture; ellipsis acts rather like a pointer or a hyperlink into memory than as an entity of its own. This conception can be related to the processing of other types of anaphors: It is not commonly assumed that in a sentence such as *The man from England drank tea, but he didn't drink coffee*, the pronoun *he* will contain the syntactic structure of the NP *the man from England* at any level of representation. Instead, an identity of *reference* between the two expressions seems to obtain (cf. Grinder and Postal, 1971, p. 269).

Note that the opposition between copying and the “memory pointer” approach is orthogonal to that between syntactic and semantic/discourse representations (cf. Phillips and Parker, 2014). Semantic representations could also be copied, just as syntactic representations could be pointed to. The processing literature has focused mainly on the copying/pointing dichotomy, even though some studies have also tested whether there is syntactic priming from ellipsis sites, with mixed results (Cai et al., 2013; Xiang et al., 2014). Murphy (1985) appears to have been the first to systematically look for effects of antecedent length on reading times for elliptical clauses, in this case the sentence *Later, his uncle did too* in (2).

(2) a. Jimmy swept the floor. Later, his uncle did too.
       b. Jimmy swept the tile floor behind the chairs free of hair and cigarettes. Later, his uncle did too.

Despite being concerned with verb-phrase ellipsis, we assume that this study is informative with regard to sluicing as well, since the most parsimonious hypothesis would be that all types of ellipsis are processed in the same way. The reasoning behind Murphy's manipulation was that “[l]onger antecedents would be expected to affect a copying process, since the longer the string that must be copied onto the anaphor, the longer it should take to understand the anaphor” (p. 293). If there was no copying, so the argument goes, then reading times for the second sentence should not differ between (2a,b). Murphy found that reading times for the elliptical sentence were increased by about 260 ms when the antecedent was long rather than short. Interestingly, this difference disappeared when another sentence was inserted between antecedent and ellipsis^2^.

The system Murphy proposes is one in which there are two processes, namely copying and discourse-based “plausible reasoning,” which operate in parallel, with the process that finishes first supplying the antecedent. When the antecedent is far away, the speed and/or availability of copying suffers and readers fall back on plausible reasoning, which by assumption is not influenced by complexity effects. Tanenhaus and Carlson (1990, p. 261) remain unconvinced by Murphy's (1985) evidence for copying, arguing that the length manipulation “also introduced potential scope and attachment ambiguities”^3^. The authors favor a pointer-based approach, while allowing for the possibility that there are both a syntax- and a discourse-based process at work.

Two additional important findings come from an experiment by Frazier and Clifton (2000) and a series of experiments by Martin and McElree (2008), all on verb-phrase ellipsis.

(3) Frazier and Clifton (2000), Experiment 1 B
       a. Sarah left her boyfriend last May. Tina did too.
       b. Sarah got up the courage to leave her boyfriend last May. Tina did too.
(4) Martin and McElree (2008), Experiment 3
       a. The history professor understood Roman mythology, …
       b. The history professor understood Rome's swift and brutal destruction of Carthage, …
         …but the principal was displeased to learn that the over-worked students attending summer session didnot.

Frazier and Clifton's study used self-paced reading and found no difference in reading times between (3a,b) for the sentence *Tina did too*. Martin and McElree's Experiment 3, which used sentences such as (4a,b), employed a speed-accuracy trade-off paradigm with end-of-sentence acceptability judgments. No effect of antecedent complexity on processing times was observed in this study and two further experiments, which the authors interpret as evidence for a pointer-based approach.

Here is where terminology becomes an issue, as Frazier and Clifton (2001) explain their earlier results by means of a mechanism called Copy α. Copy α becomes available when the scope of an ellipsis can be uniquely identified and serves as a shortcut to syntactic structure: instead of being built step-by-step, which would be computationally costly, the silent syntax is copied from the antecedent. As this process is assumed to be “cost-free,” the complexity of the copied structure has no influence on processing time. Frazier and Clifton's use of the copying metaphor is not very intuitive (cf. Martin and McElree, 2008, p. 882f.), as it should take more time to copy a larger amount of information, in concordance with Murphy (1985) prediction^4^. Indeed, Frazier and Clifton (2001, p. 17) themselves explain that a pointer would be a possible implementation of Copy α and in a later paper (Frazier and Clifton, 2005) describe Copy α as equivalent to “sharing” one structure between antecedent and ellipsis (cf. also Murguia, 2004). We will thus treat pointer-based approaches, Copy α and “sharing” as variants of one and the same idea, namely that the antecedent's structure is available in memory and can be retrieved from there as-is, without any additional costly computations.

Phillips and Parker (2014, p. 91) make note of several methodological problems in both of the above studies. Frazier and Clifton's (2000) experiment used only a small number of experimental items, all of which had the ellipsis at the very end of a sentence, where wrap-up effects might mask an influence of antecedent complexity. Additionally, comprehension questions were not asked after every trial and never targeted the interpretation of the ellipsis^5^. The ungrammatical sentences in Martin and McElree's (2008) study replaced the subject of the elliptical clause by an inanimate NP (*the overly worn books*), thus making the judgments fairly easy and possibly leading subjects to engage in superficial processing. Given these concerns, Phillips and Parker judge the results to be inconclusive, but also point out that it would be difficult to design an experiment that would provide convincing evidence for or against complexity effects.

Given this state of affairs, we think it worthwhile to look back at Frazier and Clifton's (2001) distinction between a syntactic structure that is computed step-by-step and one that is retrieved from memory. What happens when the antecedent is structured in a way that is known to fool the “normal” incremental parsing mechanism, that is, if it contains a garden path? Assuming a serial parsing architecture, recovering from a syntactic misanalysis involves reanalyzing the ambiguous region and assigning the same structure that would be computed for an unambiguous control sentence. Since the final memory representations for ambiguous and unambiguous sentences are the same, pointer-based approaches and Copy α would predict that there should be no difference in processing times at the ellipsis site. If, on the other hand, ellipsis is not resolved by linking the gap to a complete structure in memory, different scenarios are possible. One would be that the antecedent is accessed in memory as a word string, and that syntax and semantics are assigned to this string in the usual way, that is, incrementally. However, as verbatim memory is known to be highly fallible even in recognition tests (Sachs, 1967; Murphy and Shapiro, 1994), it may be unrealistic to assume that strings are *recalled* literally for ellipsis processing. The account of Kim et al. (2011) proposes that not the words themselves but their features are accessed by the parser at the ellipsis site, and that “derivations in an initial conjunct [are allowed] to do double-duty in a second conjunct” (p. 346). Their account states that “once […] an appropriate antecedent is found, [its derivation] becomes available to the parser, just as if it were located at the elision point in the input string” (p. 346), essentially claiming that the derivation is carried out twice. Now, if the sentence processor has no way of “remembering” that it was garden-pathed by the antecedent, there is a chance that it will be garden-pathed again at the ellipsis site.

A model that is, in principle, compatible with both the pointer/sharing approach and the “reparsing” account is the cue-based retrieval parser of Lewis and Vasishth (2005). In this model, syntactic phrases are stored in working memory as chunks than can be retrieved if needed. For complex phrases, both the phrase itself and its constituent parts, such as the subject of a verb phrase, are stored, along with their grammatical relations. When an ellipsis site is encountered, the parser would thus have the opportunity to retrieve either the whole antecedent as one chunk, as under a pointer-based account, or to retrieve whatever chunks are contained within the antecedent and build a new structure, as under the “reparsing” view. The latter possibility may become especially attractive in cases of antecedent-ellipsis mismatch, where a strict isomorphism condition cannot be upheld (e.g., Merchant, 2001). As in the case of Kim et al. (2011) chunks are conceived of as feature bundles and thus no verbatim memory of the antecedent is required for retrieval. In fact, both Kim et al. (2011) and Lewis and Vasishth (2005) explicitly assume that the linear order of constituents is not represented in the syntax.

The “parse twice” approach might seem counterintuitive, but is in fact no less parsimonious than Frazier and Clifton's Copy α, given that it needs no special machinery besides access to grammatical features inside the antecedent structure. One would not expect the garden-path effect at the ellipsis site to be of the same strength as the one observed for the antecedent, just as one would not expect the reading time for *when* in (1) to be equal to that of *John saw Mary*. Several steps involved in lexical access can be omitted during ellipsis processing. Simner and Smyth (1999) suggest that instead of using lexemes, ellipsis targets word lemmas, which would be compatible with the “feature bundle” view described above. Additionally, ellipsis normally occurs in environments that feature a high amount of syntactic parallelism. If a parallel structure is expected, the relevant routines may be activated beforehand or at least be assigned a higher rank when the parser decides which structure to build at the ellipsis site, which can be seen as an instance of syntactic priming (Dubey et al., 2008; Dickey and Bunger, 2011). Given this assumption, however, it might be that in case of a garden path the preferred but incorrect structure will feature into the calculation, making the ellipsis more difficult to process than in cases where the antecedent's structure is unambiguous. While Arai et al. (2014) found evidence that resolving an ambiguity in a prime sentence makes processing of the same ambiguity in the target sentence easier when the same verb is repeated (see also Branigan et al., 2005), it is unclear whether ellipsis constitutes “repetition.”

In our experiment, we used a known garden-path structure in German to test the—equivalent—predictions of pointer- and sharing-based approaches against those of a reconstruction-based approach of ellipsis processing. The former two predict that garden-pathing within the antecedent clause should have no effect at the ellipsis site while the latter predicts that the pattern observed at the point of disambiguation will reappear, although the effect size may be significantly smaller. To anticipate the results, we found an unpredicted pattern that was inconsistent with a reconstruction approach, but compatible with pointer- and sharing-based accounts if additional assumptions are made.

## 2. Materials and methods

### 2.1. Stimuli

It is known that German readers prefer to assign a subject interpretation to a sentence-initial NP that is ambiguous between a subject and an object reading, which results in a garden path when it is disambiguated toward an object role (cf. also Hemforth, 1993, among others). Different explanations for the subject preference have been proposed. For instance, Gorrell's (1996) approach assumes that the parser favors structural simplicity; under his analysis, deriving an OVS structure requires more movement operations (and thus more traces) than deriving an SVO structure, where the object presumably remains in the position at which it is base-generated. Schlesewsky et al. (2000) consider the possibility that the subject preference is due to a frequency-based “tuning” effect (e.g., Mitchell et al., 1995), reporting over 90% nominative-initial main clauses in a corpus study. Still other possibilities are that subject-first is a default parsing assumption, as has been proposed for English (e.g., Bever, 1970; Grodzinsky, 1986; Fishbein and Harris, 2014). If one follows the current standard analysis of German clause structure, where S(O)V word order is assumed to be basic and all other word orders are derived through movement (e.g., Schwartz and Vikner, 2007), the reanalysis of an object-initial structure will minimally involve removing co-indexation between an assumed trace position for the subject and the initial noun phrase, as well as postulating a trace position for an object.

The garden-path effect incurred by the non-canonical structure is stronger when disambiguation is achieved through agreement on the finite verb rather than through case marking on another NP (Meng and Bader, 2000). As shown in (5), we used indefinite NPs instead of the wh-marked NPs employed by Meng and Bader. Case marking on the *sympathizer* NP is either ambiguous (5a/b) or unambiguous (5c/d). The auxiliary *hatte(n)*, “had,” agrees either with the singular *sympathizer* or with the plural *rebels* NP, thereby signaling either OVS (5a/c) or SVO word order (5b/d). The result is a 2 × 2 design with the factors word order and case marking. Diamonds indicate the boundaries of presentation regions in the experiment, subscripts indicate region coding for the statistical analysis.

(5)   a. **Ambiguous / OVS**
             Eine Sympathisantin   der Opposition_np1_ ⋄
             A sympathizer.fem.**nom/acc** of the opposition
             hatten_aux_ ⋄ die Rebellen_np2_   ⋄   …
             **had.pl**    the rebels.nom/acc
       b. **Ambiguous / SVO**
             Eine Sympathisantin   der Opposition_np1_ ⋄
             A sympathizer.fem.**nom/acc** of the opposition
             hatte_aux_   die Rebellen_np2_   ⋄   …
             **had.sg**   the rebels.nom/acc
       c. **Unambiguous / OVS**
             Einen Sympathisanten   der Opposition_np1_ ⋄
             A sympathizer.masc.**acc**   of the opposition
             hatten_aux_ ⋄ die Rebellen_np2_   ⋄   …
             **had.pl**   the rebels.nom/acc
       d. **Unambiguous / SVO**
             Ein Sympathisant   der Opposition_np1_ ⋄
             A sympathizer.masc.**nom**   of the opposition
             hatte_aux_   ⋄ die Rebellen_np2_   ⋄   …
             **had.sg**   the rebels.nom/acc
             …laut einem Bericht_adj_ ⋄ maßgeblich unterstützt,_vp_
                according to a report   decisively   supported
             ⋄ aber ⋄ die Regierung ⋄ konnte ⋄ nicht ⋄
               but   the government   could   not
             nachweisen,_wh-1_ ⋄ wie,_wh_ ⋄ so sehr_wh+1_ ⋄ sich_wh+2_ ⋄
             substantiate   **how**   so greatly   itself
             die Untersuchungskommission_wh+3_ ⋄ auch ⋄
             the investigative commission      too
             bemühte.
             struggled
             “The rebels had supported a sympathizer (OVS, a/c)/A sympathizer had supported the rebels (SVO, b/d), but the government could not substantiate how, no matter how hard the investigative commission tried.”

The antecedent clause ends at *unterstützt*, “supported.” It is conjoined with a second clause by *aber*, “but,” which contains a sluicing site (or “sluice”) at *wie*, “how.” All wh-phrases in the experiment were “sprouted” (Chung et al., 1995), that is, they had no explicit correlate in the antecedent. We only used adjunct wh-phrases since argument wh-phrases are case-marked in German, which would have introduced a potential confound. The other wh-phrases used were several expressions meaning “why” (*warum, weshalb, wieso*), *wo*, “where,” *wann*, “when,” *womit*, “with what,” *wozu* “to what (end),” and *wobei*, “at what” (combined with the verb *unterstützen*, “to support”). The part of the sentence following the sluicing site was intended as a spillover region. We could have used only conditions (5a) and (5c) to look for an effect of reanalysis, but decided to also include (5b) and (5d) as control conditions since otherwise reanalysis would be completely confounded with the gender of the initial NP. Additionally, even though condition (5b) is initially ambiguous, there should be no reanalysis as readers will assume SVO order by default (cf. Meng and Bader, 2000); we can thus control for temporarily ambiguous antecedents being processed differently from unambiguous ones. Thirty-two sentences were created according to this schema for use in the experiment. A complete list of the experimental materials is given in the appendix. The stimuli were combined with ninety-six filler sentences featuring various constructions.

We expected a garden-path effect to occur at the auxiliary of the antecedent clause in the form of a word order × case marking interaction. Meng and Bader (2000) observed longer reaction times in a grammaticality judgment task for OVS than for SVO sentences, indicating that OVS order is overall more difficult to process. In (5a), however, the *sympathizer* NP presumably has to be reanalyzed from subject to object, which should further increase processing time. If ellipsis acts as a pointer into memory, no interaction between the experimental factors should appear at *wie*, “how,” as neither the scope of the ellipsis nor the availability of a completely analyzed antecedent structure vary between conditions. If, however, the syntax of the ellipsis site has to be constructed by normal parsing routines, the garden-path effect should reappear at this position, though most likely with reduced magnitude.

We had no specific predictions as to possible effects of OVS vs. SVO word order at the ellipsis site, but a *post-hoc* hypothesis will be developed in the discussion section. A complication concerning the predictions of both accounts that did not become apparent to us until after the experiment is that inserting a verb-second antecedent into the ellipsis site verbatim is impossible in our stimuli, as German subordinate clauses are generally required to be verb-final. The predictions outlined above are valid for well-formed antecedents, but should pertain to mismatched antecedents as well if certain additional assumptions are made, as will be explained shortly.

### 2.2. Participants

Sixty students from the University of Potsdam were recruited for the study. All subjects were native speakers of German and were either paid 6 € or received course credit for the participation. Informed consent was obtained from all participants prior to testing.

### 2.3. Procedure

The sentences were presented using the moving window self-paced reading technique (Just et al., 1982), which was implemented using the Linger software (Rohde, 2003; http://tedlab.mit.edu/~dr/Linger/). Participants sat in front of a PC in a quiet room and were instructed to read silently and at their own pace. Sentences were presented in 20 pt Courier New font according to a latin square procedure. At the beginning of each trial, all characters were masked with underscores. Participants completed two practice trials before the experiment proper. The order of fillers and experimental sentences was randomized at runtime. Each trial was followed by a comprehension test which took one of two forms: either a statement about the preceding sentence had to be judged as true or false, or a gap in a statement had to be filled by selecting one out of four options. Some test statements targeted the argument structure of the antecedent (*Rebels had supported a sympathizer of the opposition. [Yes/No]*), while others targeted other kinds of information from the sentence. The ratio of true to false statements for the judgment test was balanced. For a subset of fill-in-the-gap statements appearing after experimental sentences, participants had to supply the critical wh-pronoun^6^.

## 3. Results and discussion

### 3.1. Data analysis

After 15 participants had completed the experiment, it was noticed that three experimental items contained a typographical error in one condition each. The errors were removed and data from the corresponding trials were excluded from the statistical analysis. The remaining data were analyzed using the R software environment (R Core Team, 2015) by fitting linear mixed-effects models to individual regions of interest with the lme4 package (Bates et al., 2014). The models included varying intercepts and slopes by subjects and by items. The code and data will be released with the publication of this paper. When the estimate for a slope adjustment was zero, the random effect was dropped from the model, along with any associated higher-order effects. When a model failed to converge, random effects were removed, starting with the effect that accounted for the smallest amount of variance, until convergence was obtained. Sum contrasts were defined for the experimental factors word order and case marking and entered into the models as fixed effects. For word order, the OVS conditions were coded as 1 and the SVO conditions as −1, respectively. For case marking, the ambiguous conditions were coded as 1 and the unambiguous conditions as −1. Since processing spillover is a known concern in self-paced reading, the reading time for the immediately preceding region was also entered into all models after being appropriately transformed (see below) and subsequently centered. The addition of this parameter improved model fit for all regions of interest^7^, but the method is by no means guaranteed to eliminate spillover entirely, for instance if subjects postpone processing and keep “tapping” the button at fixed time intervals (Witzel et al., 2012).

An underlying assumption in linear modeling is that the residuals are approximately normally distributed. As this was not the case when raw reading times were used as the dependent variable, we applied the Box-Cox procedure (Box and Cox, 1964; Venables and Ripley, 2002), which suggested a reciprocal transformation (1/RT). Reciprocal reading times were multiplied by −1000 to make the parameters easier to interpret. Additionally, all data points corresponding to reading times below 150 ms were removed, which resulted in a loss of less than one per cent of data in all cases. Effects were judged as significant if *t* ≥ 2. Model output is shown in Table 2.

### 3.2. Comprehension accuracy

Participants' overall comprehension accuracy was at 90%, though accuracy for experimental items was somewhat lower at 82%. Overall, subjects were most accurate at supplying the wh-pronoun (92% accuracy) and least accurate at verifying statements about the argument structure of the antecedent (72% accuracy), with the rest of the comprehension tests falling in between (86% accuracy). All further analyses were conducted without distinguishing between question types, unless otherwise noted. A linear mixed-effects model was fit to question response times using the same procedure described above for reading times. The analysis revealed no significant effects of the experimental manipulation. An analogous model with reciprocal response time as an additional predictor was fit to response accuracies using a logit link function. The fit showed an effect of response time such that accuracy dropped with increased delay ($\hat{\beta }$ = −0.13, *se* = 0.03, *t* = −5.18), as well as a significant word order × case marking interaction ($\hat{\beta }$ = −0.18, *se* = 0.07, *t* = −2.74), which nested contrasts^8^ revealed to be driven by the OVS/ambiguous condition eliciting more incorrect responses than the SVO/ambiguous condition ($\hat{\beta }$ = −0.27, *se* = 0.13, *t* = −2.09). To investigate further, we created a new contrast between questions that queried the role of the arguments in the antecedent and questions that did not. When this distinction was entered into the model^9^, it turned out to be highly predictive of accuracy ($\hat{\beta }$ = −0.66, *se* = 0.16, *t* = −4.24), indicating that questions about argument structure were more difficult to answer than other question types. At the same time, the word order × case marking interaction was significant ($\hat{\beta }$ = −0.17, *se* = 0.07, *t* = −2.63), but there was no three−way interaction. There was thus no indication that comprehension failure for questions targeting argument structure was limited to garden-path sentences. Why answering questions about garden-path sentences should be difficult even when the temporary ambiguity is not targeted remains mysterious for the time being.

### 3.3. Reading times

**Figure 1:**
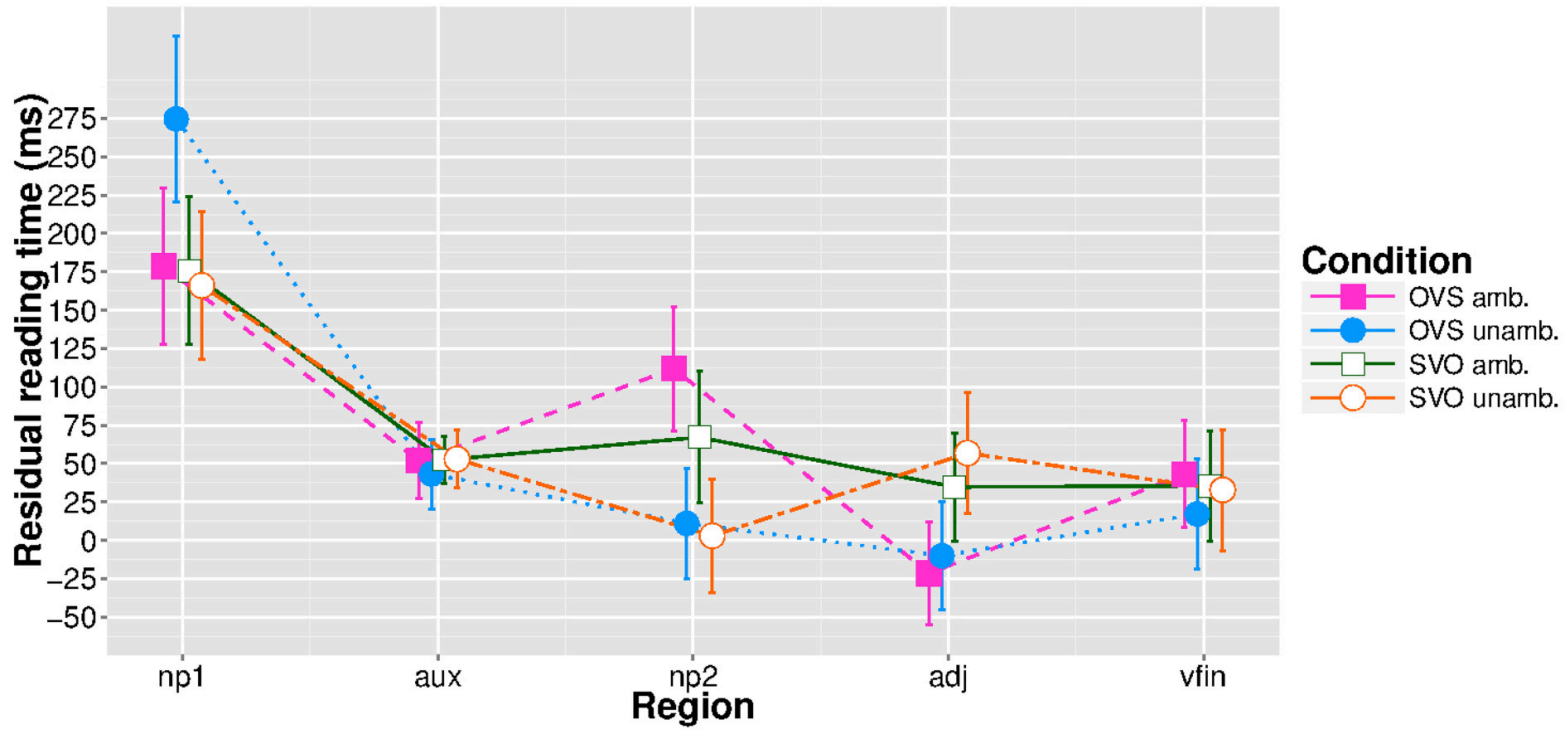
**Residual reading times for the antecedent regions, extreme values removed**. Error bars represent 95% confidence intervals.

Table 1 shows the mean raw reading times for the analyzed regions of interest. Figure 1 shows residual mean reading times for each region of the antecedent. Residualization was carried out by fitting a linear mixed-effects model with region length as a fixed effect and random slopes by subject. Unresidualized reciprocal reading times (see above) were used in the statistical analysis. A main effect of word order appeared at the auxiliary ($\hat{\beta }$ = 0.03, *se* = 0.01, *t* = 2.07), such that OVS was processed more slowly than SVO, which is likely due to the additional plural suffix in the OVS conditions. On the second NP, there were main effects of word order ($\hat{\beta }$ = 0.04, *se* = 0.01, *t* = 3.02) and case marking ($\hat{\beta }$ = 0.04, *se* = 0.01, *t* = 3.3), such that SVO was read faster than OVS and unambiguous sentences were read faster than ambiguous ones. There was also a significant interaction between the factors ($\hat{\beta }$ = 0.02, *se* = 0.01, *t* = 2.12), which nested contrasts revealed to be driven by OVS clauses taking longer to read in the presence of ambiguous case marking ($\hat{\beta }$ = 0.07, *se* = 0.02, *t* = 3.68). The preverbal adjunct again showed a main effect of word order ($\hat{\beta }$ = −0.02, *se* = 0.01, *t* = −2.38); at this position, OVS clauses were read faster than SVO clauses^10^.

**Table 1:** **Untrimmed raw mean reading times in milliseconds by condition for antecedent, ellipsis and spillover regions, standard errors in parantheses**.

		**OVS/amb**.	**SVO/amb**.	**OVS/unamb**.	**SVO/unamb**.
*A sympathizer …*	np1	1793 (48)	1760 (39)	1830 (41)	1651 (39)
*had.sg/pl*	aux	519 (17)	474 (8)	499 (12)	474 (10)
*the rebels*	np2	1021 (28)	976 (28)	913 (23)	921 (27)
*according to …*	adj	1041 (26)	1107 (29)	1066 (28)	1135 (31)
*decisively supported …*	vp	892 (23)	887 (24)	868 (22)	900 (26)
*substantiate*	wh−1	471 (8)	485 (10)	493 (9)	486 (10)
*how*	wh	423 (7)	427 (7)	422 (6)	434 (7)
*so greatly*	wh+1	437 (7)	452 (8)	449 (9)	449 (8)
*itself*	wh+2	578 (15)	564 (15)	591 (16)	584 (18)
*the …commission*	wh+3	571 (18)	580 (16)	604 (17)	590 (17)

**Table 2:** **Coefficient estimates, standard errors and ***t***-values for the linear mixed-effects models fit to reciprocal reading times at the indicated regions of interest**.

**aux**				**np2**			
	**Estimate**	**Std. Error**	***t*-value**		**Estimate**	**Std. Error**	***t*-value**
(Intercept)	−2.19	0.07	−32.82	(Intercept)	−1.32	0.08	−17.08
Case marking	0.01	0.01	0.51	Case marking	0.04	0.01	3.30
Word order	0.03	0.01	2.07	Word order	0.04	0.01	3.02
Spillover	−0.08	0.04	−1.75	Spillover	0.15	0.02	6.48
Case marking:word order	−0.01	0.01	−0.91	Case marking:word order	0.02	0.01	2.12
**adj**				**wh**+**2**			
(Intercept)	−1.17	0.07	−15.96	(Intercept)	−1.98	0.10	−20.65
Case marking	0.00	0.01	0.26	Case marking	−0.02	0.01	−1.37
Word order	−0.02	0.01	−2.38	Word order	0.03	0.01	2.02
Spillover	−0.01	0.02	−0.83	Spillover	0.25	0.02	10.22
Case marking:word order	−0.00	0.01	−0.35	Case marking:word order	0.01	0.01	0.89
**wh**+**3**			
(Intercept)	−2.11	0.10	−21.19
Case marking	−0.03	0.01	−1.86
Word order	−0.00	0.02	−0.12
Spillover	0.06	0.02	3.05
Case marking:word order	−0.03	0.01	−2.01

**Figure 2:**
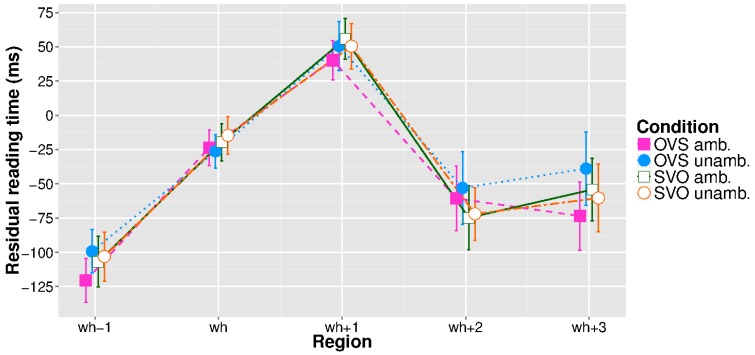
**Residual reading times for the pre-ellipsis, ellipsis, and spillover regions, extreme values removed**. Error bars represent 95% confidence intervals.

Figure 2 shows the mean reading times from the region right before the ellipsis site to three words after the ellipsis site, again in residualized form. No significant effects appeared at the wh-pronoun or in the immediately following region. In the next region (wh+2), there was a main effect of word order ($\hat{\beta }$ = 0.03, *se* = 0.01, *t* = 2.02), such that OVS clauses took longer to read than SVO clauses. For this position, closer inspection of the model revealed one very short reading time (177 ms) to be highly influential in the fit, and removing this value resulted in the effect merely approaching significance ($\hat{\beta }$ = 0.02, *se* = 0.01, *t* = 1.89). In the third region after the wh-pronoun (wh+3), a word order × case marking interaction reached significance ($\hat{\beta }$ = −0.03, *se* = 0.01, *t* = −2.02), due to the OVS/ambiguous condition being read faster than the OVS/unambiguous condition, with no single condition driving the interaction. During data analysis we noticed that five experimental sentences featured gender-marked pronouns at position wh+2, which presents a possible confound. Adding the presence vs. absence of a pronoun as a sum-coded predictor did, however, not change the results found at regions wh+2 and wh+3.

One might think that the interaction found at position wh+3 stemmed from occasional processing breakdowns in the OVS/ambiguous sentences. We assume that these would be due to failures in processing the antecedent, which would leave the parser without an adequate retrieval target for the ellipsis. To test this hypothesis, we added the reading time for the second NP, which is expected to reflect the difficulty of the garden path, to the reading time model for position wh+3 on the same trial. While this measure turned out to be a highly significant predictor ($\hat{\beta }$ = 0.13, *se* = 0.02, *t* = 5.51), the word order × case marking interaction also stayed significant and indeed became stronger ($\hat{\beta }$ = −0.03, *se* = 0.01, *t* = −2.21). This suggests that while the time spent processing the garden-path influences retrieval difficulty, there are factors above and beyond this measure which determine processing effort at the ellipsis site. In a further test, we added reading times for both the second NP and position wh+3 to the response accuracy model reported above. The reasoning behind this was that processing failure at either position could lead to incorrect responses. Adding these parameters did, however, not change the result. We also compared the median reading time in the OVS/ambiguous condition for position wh+3 with the overall median reading time for the experimental items. The difference lay within reasonable bounds (439 ms, *se* 18 ms vs. 473 ms, *se* 2 ms), indicating that very short RTs from processing failures were not pushing down the median. Congruently with this, a visual inspection of a density plot of RTs at position wh+3 did not indicate a mode or tail of fast reading times, nor did Hartigan's Dip Test (Hartigan and Hartigan, 1985) yield any evidence for bimodality. Finally, we removed all trials with incorrect responses to the comprehension test, which amounted to 18% of the data for position wh+3, and refit our model. Note that an incorrect answer does not necessarily mean that parsing failed; misinterpretations could, for instance, arise from fragments of discarded analyses in memory (see below). Nevertheless, the results of the comprehension test are the only pertinent measure available to us. With one fifth of data removed, the word order × case marking interaction stayed near the significance threshold ($\hat{\beta }$ = −0.02, *se* = 0.01, *t* = −1.62) and became marginally significant when antecedent reading time was added as a predictor ($\hat{\beta }$ = −0.03, *se* = 0.01, *t* = −1.86). The loss of significance is not particularly unexpected given the loss of statistical power incurred by removing data. To our minds, these results do not indicate that processing failure was a factor in decreasing reading times for the OVS/ambiguous condition.

### 3.4. Discussion

The expected garden-path effect for the antecedent appeared one region later than predicted, at the second NP, showing that the experimental manipulation was successful. While no effects were found at the ellipsis site itself, OVS antecedents led to longer reading times two regions downstream from the wh-pronoun. Note that this cannot be explained by a “global spillover effect” from the antecedent: earlier regions did not show the pattern, and there is no reason to assume that antecedents in the OVS/unambiguous condition were extremely difficult to process. Furthermore, an interaction between the experimental factors appeared at position wh+3, albeit in a surprising form: sentences in the OVS/ambiguous condition were read faster than those in the OVS/unambiguous condition, with the two SVO conditions lying in between. We assume that the observed pattern reflects delayed processing of the ellipsis, either as the consequence of subjects “tapping” the space bar at fixed time intervals (Witzel et al., 2012; see Discussion below) or as spillover that was not factored out by the statistical model. As the OVS/ambiguous condition was responsible for the garden-path effect within the antecedent clause, the processing advantage is unexpected with regard to the reconstruction hypothesis, which had predicted the same pattern to reappear at the ellipsis site. The result is also not straightforwardly explained by a pointer-based approach, which would have predicted no differences between the conditions. We will argue below that what we are observing at positions wh+2 and wh+3 is the interaction of two factors: antecedent-ellipsis mismatch and memory trace reactivation through reanalysis.

#### 3.4.1. German word order and antecedent-ellipsis mismatch

As we've pointed out in the introduction, German subordinate clauses are required to be verb-final^11^ while main clauses invariably have the finite verb in second position. As the sluicing structures in the present study appeared in subordinate clauses, all antecedent clauses would therefore have had to be verb-final instead of verb-second to be compatible with the gap. Given that sluicing is still perfectly acceptable in all of our stimuli, we seem to be seeing a case of “acceptable ungrammaticality” (Frazier, 2008). Both SVO and OVS antecedents were, to use the terminology of Arregui et al. (2006), “flawed,” but possibly not in the same way.

OVS order in German main clauses can be derived through topicalization, with the object occupying the so-called *Vorfeld* (“prefield,” e.g., Müller, 2005)^12^. As this strategy is not available in subordinate clauses, non-canonical word orders must be derived via *scrambling*, which moves constituents within the so-called *Mittelfeld* (“middle field,” e.g., Hinterhölzl, 2006). The slightly simplified examples in (6) illustrate this. SOV order in (6a) is unproblematic, but scrambled OSV in (6b) is, at the very least, highly marked^13^.

(6)      Die Regierung konnte nicht nachweisen,…
            the government could not substantiate
     a. **SOV subordinate clause**
         … wie die Rebellen einen Sympathisanten
             how the rebels   a sympathizer.acc
         unterstützt hatten.
         supported had.pl
     b. **OSV subordinate clause**
         … ?? wie einen Sympathisanten_i_ die Rebellen t_i_
            how a sympathizer.acc   the rebels
         unterstützt hatten.
         supported had.pl

The Recycling Hypothesis proposed by Arregui et al. (2006) predicts that ellipses are more difficult to process the more the antecedent mismatches the ellipsis site. Arregui et al. assume “repair” operations as the source of the difficulty. Assuming the verb-second antecedents have already been partly repaired by moving the verb to the end, (6b) would still need to be transformed into an SOV structure like (6a), presumably be reversing the movement operation. The increased reading times for sentences with object-initial antecedents observed at position wh+2 would be expected under the assumption that the mismatch between an OVS antecedent and an SOV sluice is greater than for SVO antecedents, where the repair process does not need to change the order of the arguments.

Two alternative suggestions made by an anonymous reviewer merit discussion. One is that the processor simply fills the ellipsis site with a verb-second clause, deriving a structure that would have no grammatical surface equivalent. There would be no reason to invoke the Recycling Hypothesis in this case, and the OVS disadvantage would need to be explained either by constraints on topicalization or possibly by invoking working memory factors. Both of these possibilities present problems. It has been found that surprising or unusual stimuli lead to better recall performance (Hirshman et al., 1989), which would lead us to expect that the more uncommon OVS antecedents should be easier instead of more difficult to retrieve. Additionally, the claim that ungrammatical structures can be derived during ellipsis processing seems extreme given that the observed effects can be explained through other means. The reviewer's second suggestion is that garden-pathing in the antecedent might result in its memory representation being more difficult to access, allowing a slower discourse-based mechanism like Murphy's (1985) to dominate during processing. However, seeing that *unambiguous* OVS antecedents also led to longer reading times at position wh+2, this does not seem like a plausible alternative to us.

#### 3.4.2. Antecedent reactivation through reanalysis

A reviewer points out that there is some evidence that initial misinterpretations of garden-path sentences persist beyond the point of disambiguation, leading to structural priming, (van Gompel et al., 2006) systematic errors during paraphrasing (Patson et al., 2009) and in comprehension tests (Christianson et al., 2001), as well as competition effects when late-arriving plausibility information contradicts the initial parse (Slattery et al., 2013). One explanation for these effects is that the initial parse of the sentence remains active in memory to some degree even after it has been discarded. In the case of our experiment, if a remnant of the discontinued subject-initial analysis remains behind in the OVS/ambiguous condition, it might be conceivable that this memory trace is considered as a possible antecedent for the ellipsis, possibly blocking access to the “real,” reanalyzed antecedent. Research on agreement processing, reflexives and subject-verb dependencies has shown that such memory interference may turn out to make processing easier or more difficult, depending on the phenomenon under study and the exact setup of the experiment (see Engelmann et al., submitted for a review). While the observed speedup in the current study may, in principle, be explained through facilitative interference, the results of Martin and McElree (2009) suggest that the availability of multiple candidate antecedents does not influence the time-course of ellipsis processing in any way. As it is unclear why the interference effect should visible in our experiment but not in theirs, we will present an alternative explanation of our results.

We suggest that the pattern at position wh+3 should be analyzed in terms of a reactivation of the antecedent's memory trace that outweighs the mismatch penalty created by the word order manipulation. As explained in the introduction section, the cue-based retrieval parser of Lewis and Vasishth (2005) incorporates the assumption that syntactic phrases are stored in working memory as chunks. If a chunk is retrieved in order to make an attachment, its activation level increases, which makes subsequent retrievals easier. A reanalysis such as the one required for sentences in the OVS/ambiguous condition should reactivate the antecedent's memory chunk as its structure needs to be changed. Later, at the ellipsis site, it should thus be retrieved faster than the other types of antecedents, to which reanalysis has not applied^14^. The mismatch effect explained above can also be accounted for through an extension of the Lewis and Vasishth (2005) model: If the wh-pronoun sets retrieval cues for a verb-final antecedent in order to match the local clausal configuration, there will be no matching chunk in memory. In order to be able to complete the retrieval, the processor may then attempt to retrieve chunks which do not match the cues perfectly, such as the main clauses in the current study. Due to the matching relative order of subject and object, an SVO chunk may resonate more strongly with the SOV cue than one with OVS word order, as schematized in (7).

(7)   a. **OVS antecedent, resonates weakly with SOV cue (O-S ≠ SO)**
               [Einen Sympathisanten hatten die Rebellen
               A   sympathizer   had.pl the rebels
               unterstützt],_OVS_ …
               supported
        b. **SVO antecedent, resonates more strongly with SOV cue (S-O ~ SO)**
               [Ein Sympathisant hatte die Rebellen
               A   sympathizer had.pl the rebels
               unterstützt],_SVO_ …
               supported
               ***wie* in subordinate clause sets SOV cue**
               … aber die Regierung konnte nicht nachweisen,
                  but the government could not substantiate
               wie [ ]_SOV_ …
                how

A lower retrieval latency would then be expected for SVO chunks, thereby predicting the observed OVS disadvantage at position wh+2^15^. The reactivation/mismatch approach is thus able to account for the observed pattern of results, but due its status as a *post-hoc* argument is in need of further empirical validation.

One might think of yet another explanation for the result, namely that reconstruction *is* taking place and that syntactic priming is responsible for the advantage in the OVS/ambiguous condition. However, such an approach would not fit with the fact that the antecedent's structure is, strictly speaking, incompatible with the word order required at the gap: As the derivations of main and subordinate clauses involve different steps, it is not obvious what exactly would be primed. One would have to make a very specific set of assumptions: First, the parser would need to blindly reconstruct the syntax of the antecedent at the ellipsis site *before* checking for possible mismatches, similarly to the anonymous reviewer's suggestion that was discussed earlier. Secondly, garden-path sentences would need to prime their final structure more strongly than unambiguous controls, which to our knowledge has not been demonstrated to date. Ambiguous/OVS antecedents would then initially gain an advantage through increased priming while both kinds of OVS antecedents would be disadvantaged during the mismatch checking phase.

#### 3.4.3. Sluicing and predictive processing

We believe that one additional result is worth mentioning, even though it was only arrived at *post-hoc*. It fits with the proposal by Yoshida et al. (2013) that predictive processing may be involved in the interpretation of sluicing structures. Yoshida et al. compared sentences in which it was either possible or impossible to analyze a specific wh-phrase as part of a sluice. The evidence suggested that as soon as the wh-phrase in question was encountered, the parser started building a sluicing structure, presumably because it is preferred over other possible continuations.

We took the implication of predictive processing as an incentive to analyze reading times for the region directly *preceding* the wh-pronoun in our own experiment: If sluicing is the preferred continuation after a wh-pronoun has been encountered, it is not unlikely that it will also rank fairly highly before that point. This is especially likely given that subordinate clauses in German require a comma, which was thus present in the pre-wh region in all of our stimuli, excluding a vast range of alternative continuations that would have been likely in Yoshida et al.'s materials.

The fitting of a linear mixed-effects model (see above) at position wh-1 revealed a significant interaction between word order and case marking ($\hat{\beta }$ = −0.03, *se* = 0.01, *t* = −2.3) which had the same sign as the one observed at position wh+3^16^. Table 3 shows the model output. However, unlike at the later position, nested contrasts showed that the interaction was driven by the OVS/unambiguous condition being read more slowly than the SVO/unambiguous condition ($\hat{\beta }$ = 0.04, *se* = 0.02, *t* = 2.24), even though the numerical pattern in raw reading times was the same as for position wh+3. We have no ready explanation for this finding. Speculatively, a heuristic may be used to estimate the fit between the sluice and the antecedent. Such a heuristic might work better when case is overtly marked, and might operate more quickly when word order is canonical. In our opinion this kind of predictive strategy makes it unlikely that processing proceeds according to the priming-based account described above, in which local constraints do not influence the initial structure assignment for the ellipsis.

**Table 3:** **Coefficient estimates, standard errors and *t*-values for the linear mixed-effects model fit to reciprocal reading times at region wh-1**.

**wh-1**			
	**Estimate**	**Std. Error**	***t*****-value**
(Intercept)	−2.10	0.06	−35.17
Case marking	−0.02	0.01	−1.60
Word order	0.01	0.01	0.93
Spillover	0.36	0.02	15.21
Case marking: word order	−0.03	0.01	−2.30

To further investigate the notion that a sluice was the expected structure in our materials, we ran a sentence completion study with thirty-five new participants. It has been suggested that the speech production system may be responsible for generating linguistic expectations in comprehension (Pickering and Garrod, 2007). As sentence continuation preferences have been shown to be predictive of processing difficulty in self-paced reading (Smith and Levy, 2011), we assume that a preference for sluicing continuations in our reading study should translate into a corresponding preference in sentence completions. The stimuli consisted of the 32 sentences used in the current reading study, along with 32 sentences from a different experiment and 96 fillers. Sentences were presented using a modified version of Linger's masked auto-paced reading (otherwise known as rapid serial visual presentation or RSVP). The stimuli from the current study were cut off right before the ellipsis site and participants were asked to complete the sentences using the first continuation that came to mind. Due to the nature of the presentation, participants could not reread the sentences while they were typing their continuation. Results showed a total of only five per cent sluicing continuations. Another 54% of continuations were non-sluiced wh-clauses, followed by *if* -clauses at seventeen per cent and *that*-clauses at seven per cent. Assuming that this pattern is not due to idiosyncrasies of the production system, the observed outcome casts some doubt on the assumption that a sluicing continuation was, in fact, highly expected in our stimuli. However, subjects in the production experiment could choose their preferred continuation freely, which may conceivably have led to more conscious deliberation on their part. It is entirely possible that sluicing is only one of several possible continuations which are pre-activated during reading, which might be enough to explain the findings of Yoshida et al. (2013) and the interaction we observed at position wh-1 in the self-paced reading study. Despite the limited scope of the production experiment, given the earlier findings by (Smith and Levy, 2011), we feel that it was important to investigate whether the predictive processing seen in comprehension maps directly onto language users' preferences in production. This is apparently not the case under the conditions tested here.

## 4. General discussion

The current experiment investigated the processing of a sluicing construction in cases where the antecedent is a garden-path structure, in this instance a clause with a subject/object ambiguity. We observed reduced reading times for sentences with garden-path antecedents three regions downstream from the ellipsis as well as directly before the ellipsis. Furthermore, there was an overall pattern of elevated reading times in the spillover region for antecedents that mismatched the canonical word order of the ellipsis site. Our results are best compatible with accounts of ellipsis resolution that can be implemented in the form of a memory pointer mechanism (Frazier and Clifton, 2001, 2005; Martin and McElree, 2008), which would need to be augmented to account for reactivation assumed by the cue-based retrieval parser of Lewis and Vasishth (2005). The evidence for a mismatch effect is in line with the predictions of the Recycling Hypothesis proposed by Arregui et al. (2006). However, given that we have observed no evidence for reconstruction in our experiment, we do not subscribe to Arregui et al.'s assumption that “flawed” antecedents are “repaired” in a way that is similar to syntactic reanalysis (p. 242). The mismatch effect may be better approached along the lines of the wh-pronoun setting a retrieval cue for an antecedent that matches the word order requirements of the local clause, opting for the closest candidate upon failure. Alternatively, one could follow the proposal of Kim et al. (2011), in which ellipses with non-canonical antecedents violate parsing heuristics that are based on construction frequency and expectation. Under an approach without reconstruction, we would claim that it is not a parsing heuristic that is violated, but a local expectation as to what an antecedent targeted by retrieval should look like. If the expectation were global, no mismatch effect would be expected, given that the antecedent has already been encountered in the input. The local expectation account fits with the pattern observed by Yoshida et al. (2013) as well as with the effect found in the pre-sluice region (wh-1) in the current study.

Still, why did we observe a pattern in which the experimental manipulation seemed to have an effect before and after, but not *at* the ellipsis site? We assume that this is due to either insufficient statistical power, to our subjects' reading strategies, or both. Power is always an issue when effect sizes are as small as in the current study: the mean reading time difference between the unambiguous/OVS and the ambiguous/OVS conditions at position wh+3 was only 30 ms. Given this value and the associated standard errors, the *post-hoc* power to detect a real effect was at 45%, which is comparable to Frazier and Clifton's (2000) study, where the computation yields 43% *post-hoc* power^17^. The bottom line is that sample size needs to be significantly increased in order to convincingly argue that there really is *no* effect of the manipulation, even though this might be construed as trying to “force significance.”

The concern related to reading strategies comes from the fact that while non-cumulative self-paced reading more closely resembles data from natural reading than the cumulative variant does (Just et al., 1982), it is by no means certain that subjects will not adopt a “wait and see” strategy at least on some trials, meaning that they will press the button at a fixed rate and only then start processing. Witzel et al. (2012), suspecting such rhythmic “tapping” in their data, tried to remove its influence by calculating the standard deviation of the response time by subject and excluding the participants with the smallest variability, which did, however, not change their statistical result. The authors conclude that either ‘tapping’ was not a factor in their data or their method was not suitable to account for it, leaving the issue for future research. We will do the same here.

There is also a slightly different explanation for the delay we observed, namely that subjects *did* process the words the words as they were revealed, but postponed the processing of the ellipsis until they had more information. Such a strategy might make sense considering that an embedded question (i.e., an interrogative clause that serves as a complement, as in …*, but the government could not substantiate how, …*) in itself usually imparts no relevant information apart from the fact that some piece of information is missing. As the contents of the spillover region put this information in context (…*, because/so that/even though/until …*), the relevance may have become apparent, causing the observed processing pattern.

A final objection to our study would be that there was no control condition without ellipsis. It should be noted that it is extremely difficult to create closely matched controls for our sentences, given that possible continuations are limited to complement clauses, which usually feature more than one word. Other studies on ellipsis processing also lack controls [e.g., Frazier and Clifton, 2000, 2005 (except Experiments 2 and 3), Poirier et al., 2010], leaving open the possibility that any observed effects do not actually stem from the antecedent being recovered due to a perceived gap in the sentence but from some other mechanism. While this criticism can be met by pointing to the localization of the effects, as well as to the unavailability of a plausible alternative explanation, it would be desirable to include controls in future studies to strengthen the conclusions drawn from the data.

Further investigations into the interaction between antecedent ambiguity and ellipsis processing are already underway in our laboratory. We are currently aiming to find further evidence for the reactivation effect using different kinds of temporary ambiguities and ellipses, as well as experimental procedures other than self-paced reading (e.g., eye tracking).

## Funding

This research was funded by the University of Potsdam.

### Conflict of interest statement

The author declares that the research was conducted in the absence of any commercial or financial relationships that could be construed as a potential conflict of interest.

## Footnotes

^1^There is no condition of strict identity, however, as several kinds of mismatch can be observed, as in *The car was supposed to be washed but nobody did* (e.g., Kertz, 2000; Merchant, 2013, submitted).

^2^Murphy was concerned that the observed complexity effect was simply due to processing spillover from the antecedent sentence into the ellipsis sentence, but the intervening sentence did not show any effects either.

^3^It is not obvious which ambiguities the authors are referring to, or how they would impact processing under an approach without copying. It should be pointed out, however, that interpreting the ellipsis with the long antecedent in (2) requires an additional assumption, namely that the floor became dirty again between the first and the second sweeping.

^4^A possible analogy would be the copying of a file from one location on a hard drive to another, which becomes more time-consuming as file size increases.

^5^An anonymous reviewer suggests that this might have been in order not to risk making the subjects aware of the experimental manipulation. While this is a fair point, it has been shown that subjects adapt their processing strategy to task demands, trying to minimize effort through underspecification (e.g., Foertsch and Gernsbacher, 1994; Swets et al., 2008). If one intends to investigate “deep” processing, we believe that the latter risk outweighs the former, being aware that the opposite stance is equally tenable.

^6^Though the specific example in (5) was not accompanied by this kind of test, a possible fill-the-gap statement could have been *The government could not substantiate* ____ *rebels had supported a sympathizer of the opposition. [why/how/when/if]*.

^7^Improvement of fit was assessed through likelihood ratio tests comparing models with and without the spillover predictor.

^8^For this analysis, case marking was treated as nested within word order. One sum contrast compared the two ambiguous conditions, one compared the two unambiguous conditions, and a third one the OVS vs. SVO conditions.

^9^The fixed effect of reciprocal response time was removed from this model as it consistently led to convergence failure.

^10^Speculatively, this effect may be due to readers trying to make up for lost time after having been slowed down.

^11^The only exception to this rule occurs when the verb takes a sentential complement, which was not the case in our experiment.

^12^The *Feldertheorie* of German sentence structure was first developed by Drach (1937), and is also known as the *Topological Model*.

^13^Apart from not being licensed by information structure, moving the object in (6b) also violates a constraint dictating that definite noun phrases should appear before indefinite ones (see Müller, 1999 for an optimality-theoretic account).

^14^This presupposes that trace decay has not reduced the activation of the antecedent to zero in any case by the time the ellipsis is processed. The model of Lewis and Vasishth (2005) assumes that the activation of chunks than have been reaccessed is higher even after complete decay.

^15^In order to derive grammatical structures, repair processes that change the word order to verb-final would still need to apply after retrieval.

^16^As a sanity check, we also analyzed reading times at position wh-2, finding no significant effects.

^17^Note that this is not the *true* power of the experiments, which depends on the unknown true effect size.

## Appendix - Experimental materials

Eine Vertreterin der Gewerkschaft ^*^ hatten ^*^ die anwesenden Minister ^*^ während der Sitzung ^*^ scharf attackiert, ^*^ aber ^*^ der gesprächige Parlamentarier ^*^ wusste ^*^ selbst ^*^ nicht, ^*^ warum, ^*^ denn ^*^ er ^*^ war ^*^ nicht ^*^ dabei gewesen.

Eine Vertreterin der Gewerkschaft ^*^ hatte ^*^ die anwesenden Minister ^*^ während der Sitzung ^*^ scharf attackiert, ^*^ aber ^*^ der gesprächige Parlamentarier ^*^ wusste ^*^ selbst ^*^ nicht, ^*^ warum, ^*^ denn ^*^ er ^*^ war ^*^ nicht ^*^ dabei gewesen.

Einen Vertreter der Gewerkschaft ^*^ hatten ^*^ die anwesenden Minister ^*^ während der Sitzung ^*^ scharf attackiert, ^*^ aber ^*^ der gesprächige Parlamentarier ^*^ wusste ^*^ selbst ^*^ nicht, ^*^ warum, ^*^ denn ^*^ er ^*^ war ^*^ nicht ^*^ dabei gewesen.

Ein Vertreter der Gewerkschaft ^*^ hatte ^*^ die anwesenden Minister ^*^ während der Sitzung ^*^ scharf attackiert, ^*^ aber ^*^ der gesprächige Parlamentarier ^*^ wusste ^*^ selbst ^*^ nicht, ^*^ warum, ^*^ denn ^*^ er ^*^ war ^*^ nicht ^*^ dabei gewesen. Eine Vertraute des Bürgermeisters ^*^ hatten ^*^ die Ratsmitglieder ^*^ kurz vor der Wahl ^*^ auffallend häufig angerufen, ^*^ aber ^*^ heute ^*^ weiß ^*^ niemand ^*^ mehr, ^*^ warum, ^*^ wie ^*^ eine Zeitung ^*^ kürzlich ^*^ in einem Kommentar ^*^ schrieb.

Eine Vertraute des Bürgermeisters ^*^ hatte ^*^ die Ratsmitglieder ^*^ kurz vor der Wahl ^*^ auffallend häufig angerufen, ^*^ aber ^*^ heute ^*^ weiß ^*^ niemand ^*^ mehr, ^*^ warum, ^*^ wie ^*^ eine Zeitung ^*^ kürzlich ^*^ in einem Kommentar ^*^ schrieb. Einen Vertrauten des Bürgermeisters ^*^ hatten ^*^ die Ratsmitglieder ^*^ kurz vor der Wahl ^*^ auffallend häufig angerufen, ^*^ aber ^*^ heute ^*^ weiß ^*^ niemand ^*^ mehr, ^*^ warum, ^*^ wie ^*^ eine Zeitung ^*^ kürzlich ^*^ in einem Kommentar ^*^ schrieb. Ein Vertrauter des Bürgermeisters ^*^ hatte ^*^ die Ratsmitglieder ^*^ kurz vor der Wahl ^*^ auffallend häufig angerufen, ^*^ aber ^*^ heute ^*^ weiß ^*^ niemand ^*^ mehr, ^*^ warum, ^*^ wie ^*^ eine Zeitung ^*^ kürzlich ^*^ in einem Kommentar ^*^ schrieb. Eine Kellnerin des Lokals ^*^ hatten ^*^ die Stammgäste ^*^ über das geplante Skatturnier ^*^ ausgefragt, ^*^ aber ^*^ der Wirt ^*^ konnte ^*^ nicht ^*^ sagen, ^*^ warum, ^*^ da ^*^ er ^*^ offenbar ^*^ an jenem Abend ^*^ sehr beschäftigt gewesen war.

Eine Kellnerin des Lokals ^*^ hatte ^*^ die Stammgäste ^*^ über das geplante Skatturnier ^*^ ausgefragt, ^*^ aber ^*^ der Wirt ^*^ konnte ^*^ nicht ^*^ sagen, ^*^ warum, ^*^ da ^*^ er ^*^ offenbar ^*^ an jenem Abend ^*^ sehr beschäftigt gewesen war.

Einen Kellner des Lokals ^*^ hatten ^*^ die Stammgäste ^*^ über das geplante Skatturnier ^*^ ausgefragt, ^*^ aber ^*^ der Wirt ^*^ konnte ^*^ nicht ^*^ sagen, ^*^ warum, ^*^ da ^*^ er ^*^ offenbar ^*^ an jenem Abend ^*^ sehr beschäftigt gewesen war. Ein Kellner des Lokals ^*^ hatte ^*^ die Stammgäste ^*^ über das geplante Skatturnier ^*^ ausgefragt, ^*^ aber ^*^ der Wirt ^*^ konnte ^*^ nicht ^*^ sagen, ^*^ warum, ^*^ da ^*^ er ^*^ offenbar ^*^ an jenem Abend ^*^ sehr beschäftigt gewesen war.

Eine Beraterin des Präsidenten ^*^ hatten ^*^ die Ermittler ^*^ offensichtlich ^*^ mit Erfolg getäuscht, ^*^ aber ^*^ man ^*^ fand ^*^ nie ^*^ heraus, ^*^ wie, ^*^ denn ^*^ es ^*^ galt ^*^ nach wie vor ^*^ die höchste Geheimhaltungsstufe.

Eine Beraterin des Präsidenten ^*^ hatte ^*^ die Ermittler ^*^ offensichtlich ^*^ mit Erfolg getäuscht, ^*^ aber ^*^ man ^*^ fand ^*^ nie ^*^ heraus, ^*^ wie, ^*^ denn ^*^ es ^*^ galt ^*^ nach wie vor ^*^ die höchste Geheimhaltungsstufe.

Einen Berater des Präsidenten ^*^ hatten ^*^ die Ermittler ^*^ offensichtlich ^*^ mit Erfolg getäuscht, ^*^ aber ^*^ man ^*^ fand ^*^ nie ^*^ heraus, ^*^ wie, ^*^ denn ^*^ es ^*^ galt ^*^ nach wie vor ^*^ die höchste Geheimhaltungsstufe.

Ein Berater des Präsidenten ^*^ hatte ^*^ die Ermittler ^*^ offensichtlich ^*^ mit Erfolg getäuscht, ^*^ aber ^*^ man ^*^ fand ^*^ nie ^*^ heraus, ^*^ wie, ^*^ denn ^*^ es ^*^ galt ^*^ nach wie vor ^*^ die höchste Geheimhaltungsstufe.

Eine Sprecherin des Pharmakonzerns ^*^ hatten ^*^ die Sportler ^*^ nach Angaben der Presse ^*^ persönlich getroffen, ^*^ aber ^*^ die Quelle ^*^ konnte ^*^ nicht ^*^ mitteilen, ^*^ wo, ^*^ sodass ^*^ die Geschichte ^*^ den meisten Lesern ^*^ wahrscheinlich ^*^ nicht sehr glaubwürdig erschien.

Eine Sprecherin des Pharmakonzerns ^*^ hatte ^*^ die Sportler ^*^ nach Angaben der Presse ^*^ persönlich getroffen, ^*^ aber ^*^ die Quelle ^*^ konnte ^*^ nicht ^*^ mitteilen, ^*^ wo, ^*^ sodass ^*^ die Geschichte ^*^ den meisten Lesern ^*^ wahrscheinlich ^*^ nicht sehr glaubwürdig erschien.

Einen Sprecher des Pharmakonzerns ^*^ hatten ^*^ die Sportler ^*^ nach Angaben der Presse ^*^ persönlich getroffen, ^*^ aber ^*^ die Quelle ^*^ konnte ^*^ nicht ^*^ mitteilen, ^*^ wo, ^*^ sodass ^*^ die Geschichte ^*^ den meisten Lesern ^*^ wahrscheinlich ^*^ nicht sehr glaubwürdig erschien.

Ein Sprecher des Pharmakonzerns ^*^ hatte ^*^ die Sportler ^*^ nach Angaben der Presse ^*^ persönlich getroffen, ^*^ aber ^*^ die Quelle ^*^ konnte ^*^ nicht ^*^ mitteilen, ^*^ wo, ^*^ sodass ^*^ die Geschichte ^*^ den meisten Lesern ^*^ wahrscheinlich ^*^ nicht sehr glaubwürdig erschien.

Eine Sympathisantin der Opposition ^*^ hatten ^*^ die Rebellen ^*^ laut einem Bericht ^*^ maßgeblich unterstützt, ^*^ aber ^*^ die Regierung ^*^ konnte ^*^ nicht ^*^ nachweisen, ^*^ wie, ^*^ so sehr ^*^ sich ^*^ die Untersuchungskommission ^*^ auch ^*^ bemühte.

Eine Sympathisantin der Opposition ^*^ hatte ^*^ die Rebellen ^*^ laut einem Bericht ^*^ maßgeblich unterstützt, ^*^ aber ^*^ die Regierung ^*^ konnte ^*^ nicht ^*^ nachweisen, ^*^ wie, ^*^ so sehr ^*^ sich ^*^ die Untersuchungskommission ^*^ auch ^*^ bemühte.

Einen Sympathisanten der Opposition ^*^ hatten ^*^ die Rebellen ^*^ laut einem Bericht ^*^ maßgeblich unterstützt, ^*^ aber ^*^ die Regierung ^*^ konnte ^*^ nicht ^*^ nachweisen, ^*^ wie, ^*^ so sehr ^*^ sich ^*^ die Untersuchungskommission ^*^ auch ^*^ bemühte.

Ein Sympathisant der Opposition ^*^ hatte ^*^ die Rebellen ^*^ laut einem Bericht ^*^ maßgeblich unterstützt, ^*^ aber ^*^ die Regierung ^*^ konnte ^*^ nicht ^*^ nachweisen, ^*^ wie, ^*^ so sehr ^*^ sich ^*^ die Untersuchungskommission ^*^ auch ^*^ bemühte.

Eine Gönnerin des Künstlers ^*^ hatten ^*^ die etwas seltsamen Verwandten ^*^ zu Anfang ^*^ des Mordes verdächtigt, ^*^ aber ^*^ aus den Tagebüchern ^*^ geht ^*^ nicht ^*^ hervor, ^*^ warum, ^*^ zumal ^*^ es ^*^ sich ^*^ relativ eindeutig ^*^ um Suizid handelte.

Eine Gönnerin des Künstlers ^*^ hatte ^*^ die etwas seltsamen Verwandten ^*^ zu Anfang ^*^ des Mordes verdächtigt, ^*^ aber ^*^ aus den Tagebüchern ^*^ geht ^*^ nicht ^*^ hervor, ^*^ warum, ^*^ zumal ^*^ es ^*^ sich ^*^ relativ eindeutig ^*^ um Suizid handelte.

Einen Gönner des Künstlers ^*^ hatten ^*^ die etwas seltsamen Verwandten ^*^ zu Anfang ^*^ des Mordes verdächtigt, ^*^ aber ^*^ aus den Tagebüchern ^*^ geht ^*^ nicht ^*^ hervor, ^*^ warum, ^*^ zumal ^*^ es ^*^ sich ^*^ relativ eindeutig ^*^ um Suizid handelte.

Ein Gönner des Künstlers ^*^ hatte ^*^ die etwas seltsamen Verwandten ^*^ zu Anfang ^*^ des Mordes verdächtigt, ^*^ aber ^*^ aus den Tagebüchern ^*^ geht ^*^ nicht ^*^ hervor, ^*^ warum, ^*^ zumal ^*^ es ^*^ sich ^*^ relativ eindeutig ^*^ um Suizid handelte.

Eine Schülerin des Schachmeisters ^*^ hatten ^*^ die Schiedsrichter ^*^ während des Turniers ^*^ sehr genau beobachtet, ^*^ aber ^*^ der aufmerksame Zuschauer ^*^ fragte ^*^ sich ^*^ noch immer, ^*^ warum, ^*^ als ^*^ er ^*^ am Abend ^*^ endlich ^*^ nach Hause kam.

Eine Schülerin des Schachmeisters ^*^ hatte ^*^ die Schiedsrichter ^*^ während des Turniers ^*^ sehr genau beobachtet, ^*^ aber ^*^ der aufmerksame Zuschauer ^*^ fragte ^*^ sich ^*^ noch immer, ^*^ warum, ^*^ als ^*^ er ^*^ am Abend ^*^ endlich ^*^ nach Hause kam.

Einen Schüler des Schachmeisters ^*^ hatten ^*^ die Schiedsrichter ^*^ während des Turniers ^*^ sehr genau beobachtet, ^*^ aber ^*^ der aufmerksame Zuschauer ^*^ fragte ^*^ sich ^*^ noch immer, ^*^ warum, ^*^ als ^*^ er ^*^ am Abend ^*^ endlich ^*^ nach Hause kam.

Ein Schüler des Schachmeisters ^*^ hatte ^*^ die Schiedsrichter ^*^ während des Turniers ^*^ sehr genau beobachtet, ^*^ aber ^*^ der aufmerksame Zuschauer ^*^ fragte ^*^ sich ^*^ noch immer, ^*^ warum, ^*^ als ^*^ er ^*^ am Abend ^*^ endlich ^*^ nach Hause kam.

Eine Spielerin des Vereins ^*^ hatten ^*^ die aufdringlichen Fans ^*^ nach dem Auswärtsspiel ^*^ grob beleidigt, ^*^ aber ^*^ der Trainer ^*^ konnte ^*^ nicht ^*^ verstehen, ^*^ warum, ^*^ sodass ^*^ er ^*^ nur ^*^ enttäuscht ^*^ den Kopf schüttelte.

Eine Spielerin des Vereins ^*^ hatte ^*^ die aufdringlichen Fans ^*^ nach dem Auswärtsspiel ^*^ grob beleidigt, ^*^ aber ^*^ der Trainer ^*^ konnte ^*^ nicht ^*^ verstehen, ^*^ warum, ^*^ sodass ^*^ er ^*^ nur ^*^ enttäuscht ^*^ den Kopf schüttelte.

Einen Spieler des Vereins ^*^ hatten ^*^ die aufdringlichen Fans ^*^ nach dem Auswärtsspiel ^*^ grob beleidigt, ^*^ aber ^*^ der Trainer ^*^ konnte ^*^ nicht ^*^ verstehen, ^*^ warum, ^*^ sodass ^*^ er ^*^ nur ^*^ enttäuscht ^*^ den Kopf schüttelte.

Ein Spieler des Vereins ^*^ hatte ^*^ die aufdringlichen Fans ^*^ nach dem Auswärtsspiel ^*^ grob beleidigt, ^*^ aber ^*^ der Trainer ^*^ konnte ^*^ nicht ^*^ verstehen, ^*^ warum, ^*^ sodass ^*^ er ^*^ nur ^*^ enttäuscht ^*^ den Kopf schüttelte.

Eine Geschworene des Gerichts ^*^ hatten ^*^ die beiden Angeklagten ^*^ trotz richterlicher Verwarnung ^*^ direkt angesprochen, ^*^ aber ^*^ niemand im Saal ^*^ verstand ^*^ wohl ^*^ so recht, ^*^ weshalb, ^*^ bevor ^*^ die Verhandlung ^*^ überraschend ^*^ auf unbestimmte Zeit ^*^ vertagt wurde.

Eine Geschworene des Gerichts ^*^ hatte ^*^ die beiden Angeklagten ^*^ trotz richterlicher Verwarnung ^*^ direkt angesprochen, ^*^ aber ^*^ niemand im Saal ^*^ verstand ^*^ wohl ^*^ so recht, ^*^ weshalb, ^*^ bevor ^*^ die Verhandlung ^*^ überraschend ^*^ auf unbestimmte Zeit ^*^ vertagt wurde.

Einen Geschworenen des Gerichts ^*^ hatten ^*^ die beiden Angeklagten ^*^ trotz richterlicher Verwarnung ^*^ direkt angesprochen, ^*^ aber ^*^ niemand im Saal ^*^ verstand ^*^ wohl ^*^ so recht, ^*^ weshalb, ^*^ bevor ^*^ die Verhandlung ^*^ überraschend ^*^ auf unbestimmte Zeit ^*^ vertagt wurde.

Ein Geschworener des Gerichts ^*^ hatte ^*^ die beiden Angeklagten ^*^ trotz richterlicher Verwarnung ^*^ direkt angesprochen, ^*^ aber ^*^ niemand im Saal ^*^ verstand ^*^ wohl ^*^ so recht, ^*^ weshalb, ^*^ bevor ^*^ die Verhandlung ^*^ überraschend ^*^ auf unbestimmte Zeit ^*^ vertagt wurde.

Eine Mitarbeiterin der maroden Firma ^*^ hatten ^*^ die Geschäftsführer ^*^ in das raffinierte Veruntreuungssystem ^*^ eingeweiht, ^*^ aber ^*^ es ^*^ herrscht ^*^ Uneinigkeit ^*^ darüber, ^*^ wann, ^*^ denn ^*^ von den belastenden Dokumenten ^*^ trägt ^*^ keines ^*^ ein Datum.

Eine Mitarbeiterin der maroden Firma ^*^ hatte ^*^ die Geschäftsführer ^*^ in das raffinierte Veruntreuungssystem ^*^ eingeweiht, ^*^ aber ^*^ es ^*^ herrscht ^*^ Uneinigkeit ^*^ darüber, ^*^ wann, ^*^ denn ^*^ von den belastenden Dokumenten ^*^ trägt ^*^ keines ^*^ ein Datum.

Einen Mitarbeiter der maroden Firma ^*^ hatten ^*^ die Geschäftsführer ^*^ in das raffinierte Veruntreuungssystem ^*^ eingeweiht, ^*^ aber ^*^ es ^*^ herrscht ^*^ Uneinigkeit ^*^ darüber, ^*^ wann, ^*^ denn ^*^ von den belastenden Dokumenten ^*^ trägt ^*^ keines ^*^ ein Datum.

Ein Mitarbeiter der maroden Firma ^*^ hatte ^*^ die Geschäftsführer ^*^ in das raffinierte Veruntreuungssystem ^*^ eingeweiht, ^*^ aber ^*^ es ^*^ herrscht ^*^ Uneinigkeit ^*^ darüber, ^*^ wann, ^*^ denn ^*^ von den belastenden Dokumenten ^*^ trägt ^*^ keines ^*^ ein Datum.

Eine Aufseherin des Gefängnisses ^*^ hatten ^*^ die verdächtigen Häftlinge ^*^ durch ein erfundenes Alibi ^*^ gedeckt, ^*^ aber ^*^ keinem der Beteiligten ^*^ war ^*^ damals ^*^ zu entlocken, ^*^ wieso, ^*^ denn ^*^ eine Aussage ^*^ hätte ^*^ wohl ^*^ gegen die Ehre verstoßen.

Eine Aufseherin des Gefängnisses ^*^ hatte ^*^ die verdächtigen Häftlinge ^*^ durch ein erfundenes Alibi ^*^ gedeckt, ^*^ aber ^*^ keinem der Beteiligten ^*^ war ^*^ damals ^*^ zu entlocken, ^*^ wieso, ^*^ denn ^*^ eine Aussage ^*^ hätte ^*^ wohl ^*^ gegen die Ehre verstoßen.

Einen Aufseher des Gefängnisses ^*^ hatten ^*^ die verdächtigen Häftlinge ^*^ durch ein erfundenes Alibi ^*^ gedeckt, ^*^ aber ^*^ keinem der Beteiligten ^*^ war ^*^ damals ^*^ zu entlocken, ^*^ wieso, ^*^ denn ^*^ eine Aussage ^*^ hätte ^*^ wohl ^*^ gegen die Ehre verstoßen.

Ein Aufseher des Gefängnisses ^*^ hatte ^*^ die verdächtigen Häftlinge ^*^ durch ein erfundenes Alibi ^*^ gedeckt, ^*^ aber ^*^ keinem der Beteiligten ^*^ war ^*^ damals ^*^ zu entlocken, ^*^ wieso, ^*^ denn ^*^ eine Aussage ^*^ hätte ^*^ wohl ^*^ gegen die Ehre verstoßen.

Eine Angestellte des städtischen Verkehrsunternehmens ^*^ hatten ^*^ die Fahrgäste ^*^ mit unverschämten äußerungen ^*^ belästigt, ^*^ aber ^*^ das Team von Soziologen ^*^ konnte ^*^ nicht ^*^ erklären, ^*^ wieso, ^*^ sodass ^*^ der Zwischenfall ^*^ für die Wissenschaft ^*^ bis heute ^*^ rätselhaft bleibt.

Eine Angestellte des städtischen Verkehrsunternehmens ^*^ hatte ^*^ die Fahrgäste ^*^ mit unverschämten äußerungen ^*^ belästigt, ^*^ aber ^*^ das Team von Soziologen ^*^ konnte ^*^ nicht ^*^ erklären, ^*^ wieso, ^*^ sodass ^*^ der Zwischenfall ^*^ für die Wissenschaft ^*^ bis heute ^*^ rätselhaft bleibt.

Einen Angestellten des städtischen Verkehrsunternehmens ^*^ hatten ^*^ die Fahrgäste ^*^ mit unverschämten äußerungen ^*^ belästigt, ^*^ aber ^*^ das Team von Soziologen ^*^ konnte ^*^ nicht ^*^ erklären, ^*^ wieso, ^*^ sodass ^*^ der Zwischenfall ^*^ für die Wissenschaft ^*^ bis heute ^*^ rätselhaft bleibt.

Ein Angestellter des städtischen Verkehrsunternehmens ^*^ hatte ^*^ die Fahrgäste ^*^ mit unverschämten äußerungen ^*^ belästigt, ^*^ aber ^*^ das Team von Soziologen ^*^ konnte ^*^ nicht ^*^ erklären, ^*^ wieso, ^*^ sodass ^*^ der Zwischenfall ^*^ für die Wissenschaft ^*^ bis heute ^*^ rätselhaft bleibt.

Eine Dolmetscherin des Botschafters ^*^ hatten ^*^ die Gastgeber ^*^ während der Begrüßungszeremonie ^*^ empfindlich gekränkt, ^*^ aber ^*^ damals ^*^ konnte ^*^ niemand ^*^ nachvollziehen, ^*^ womit, ^*^ obwohl ^*^ die kulturellen Gepflogenheiten ^*^ der jeweils anderen Seite ^*^ auf jeden Fall ^*^ hinreichend bekannt waren.

Eine Dolmetscherin des Botschafters ^*^ hatte ^*^ die Gastgeber ^*^ während der Begrüßungszeremonie ^*^ empfindlich gekränkt, ^*^ aber ^*^ damals ^*^ konnte ^*^ niemand ^*^ nachvollziehen, ^*^ womit, ^*^ obwohl ^*^ die kulturellen Gepflogenheiten ^*^ der jeweils anderen Seite ^*^ auf jeden Fall ^*^ hinreichend bekannt waren.

Einen Dolmetscher des Botschafters ^*^ hatten ^*^ die Gastgeber ^*^ während der Begrüßungszeremonie ^*^ empfindlich gekränkt, ^*^ aber ^*^ damals ^*^ konnte ^*^ niemand ^*^ nachvollziehen, ^*^ womit, ^*^ obwohl ^*^ die kulturellen Gepflogenheiten ^*^ der jeweils anderen Seite ^*^ auf jeden Fall ^*^ hinreichend bekannt waren.

Ein Dolmetscher des Botschafters ^*^ hatte ^*^ die Gastgeber ^*^ während der Begrüßungszeremonie ^*^ empfindlich gekränkt, ^*^ aber ^*^ damals ^*^ konnte ^*^ niemand ^*^ nachvollziehen, ^*^ womit, ^*^ obwohl ^*^ die kulturellen Gepflogenheiten ^*^ der jeweils anderen Seite ^*^ auf jeden Fall ^*^ hinreichend bekannt waren.

Eine Spionin des Inlandsgeheimdienstes ^*^ hatten ^*^ die Informanten ^*^ im Vorfeld der Verhandlungen ^*^ enttarnt, ^*^ aber ^*^ nicht einmal Experten ^*^ wussten ^*^ letztlich ^*^ zu sagen, ^*^ wie, ^*^ bis ^*^ irgendwann ^*^ eine Reinigungskraft ^*^ im Schutz der Anonymität ^*^ den entscheidenden Hinweis gab.

Eine Spionin des Inlandsgeheimdienstes ^*^ hatte ^*^ die Informanten ^*^ im Vorfeld der Verhandlungen ^*^ enttarnt, ^*^ aber ^*^ nicht einmal Experten ^*^ wussten ^*^ letztlich ^*^ zu sagen, ^*^ wie, ^*^ bis ^*^ irgendwann ^*^ eine Reinigungskraft ^*^ im Schutz der Anonymität ^*^ den entscheidenden Hinweis gab.

Einen Spion des Inlandsgeheimdienstes ^*^ hatten ^*^ die Informanten ^*^ im Vorfeld der Verhandlungen ^*^ enttarnt, ^*^ aber ^*^ nicht einmal Experten ^*^ wussten ^*^ letztlich ^*^ zu sagen, ^*^ wie, ^*^ bis ^*^ irgendwann ^*^ eine Reinigungskraft ^*^ im Schutz der Anonymität ^*^ den entscheidenden Hinweis gab.

Ein Spion des Inlandsgeheimdienstes ^*^ hatte ^*^ die Informanten ^*^ im Vorfeld der Verhandlungen ^*^ enttarnt, ^*^ aber ^*^ nicht einmal Experten ^*^ wussten ^*^ letztlich ^*^ zu sagen, ^*^ wie, ^*^ bis ^*^ irgendwann ^*^ eine Reinigungskraft ^*^ im Schutz der Anonymität ^*^ den entscheidenden Hinweis gab.

Eine Redakteurin der Tageszeitung ^*^ hatten ^*^ die maskierten Aktivisten ^*^ zu einer geheimen Videokonferenz ^*^ eingeladen, ^*^ aber ^*^ niemand ^*^ konnte ^*^ überzeugend ^*^ begründen, ^*^ wieso, ^*^ nachdem ^*^ das Vorhaben ^*^ unbeabsichtigterweise ^*^ der Öffentlichkeit ^*^ bekannt geworden war.

Eine Redakteurin der Tageszeitung ^*^ hatte ^*^ die maskierten Aktivisten ^*^ zu einer geheimen Videokonferenz ^*^ eingeladen, ^*^ aber ^*^ niemand ^*^ konnte ^*^ überzeugend ^*^ begründen, ^*^ wieso, ^*^ nachdem ^*^ das Vorhaben ^*^ unbeabsichtigterweise ^*^ der Öffentlichkeit ^*^ bekannt geworden war.

Einen Redakteur der Tageszeitung ^*^ hatten ^*^ die maskierten Aktivisten ^*^ zu einer geheimen Videokonferenz ^*^ eingeladen, ^*^ aber ^*^ niemand ^*^ konnte ^*^ überzeugend ^*^ begründen, ^*^ wieso, ^*^ nachdem ^*^ das Vorhaben ^*^ unbeabsichtigterweise ^*^ der Öffentlichkeit ^*^ bekannt geworden war.

Ein Redakteur der Tageszeitung ^*^ hatte ^*^ die maskierten Aktivisten ^*^ zu einer geheimen Videokonferenz ^*^ eingeladen, ^*^ aber ^*^ niemand ^*^ konnte ^*^ überzeugend ^*^ begründen, ^*^ wieso, ^*^ nachdem ^*^ das Vorhaben ^*^ unbeabsichtigterweise ^*^ der Öffentlichkeit ^*^ bekannt geworden war.

Eine Sachverständige aus Osteuropa ^*^ hatten ^*^ die Investoren ^*^ in der Planungsphase ^*^ eigenständig hinzugezogen, ^*^ aber ^*^ im Nachhinein ^*^ fragte ^*^ sich ^*^ so mancher Gutachter, ^*^ wieso, ^*^ da ^*^ das Ergebnis ^*^ augenscheinlich ^*^ nicht ^*^ verbessert wurde.

Eine Sachverständige aus Osteuropa ^*^ hatte ^*^ die Investoren ^*^ in der Planungsphase ^*^ eigenständig hinzugezogen, ^*^ aber ^*^ im Nachhinein ^*^ fragte ^*^ sich ^*^ so mancher Gutachter, ^*^ wieso, ^*^ da ^*^ das Ergebnis ^*^ augenscheinlich ^*^ nicht ^*^ verbessert wurde.

Einen Sachverständigen aus Osteuropa ^*^ hatten ^*^ die Investoren ^*^ in der Planungsphase ^*^ eigenständig hinzugezogen, ^*^ aber ^*^ im Nachhinein ^*^ fragte ^*^ sich ^*^ so mancher Gutachter, ^*^ wieso, ^*^ da ^*^ das Ergebnis ^*^ augenscheinlich ^*^ nicht ^*^ verbessert wurde.

Ein Sachverständiger aus Osteuropa ^*^ hatte ^*^ die Investoren ^*^ in der Planungsphase ^*^ eigenständig hinzugezogen, ^*^ aber ^*^ im Nachhinein ^*^ fragte ^*^ sich ^*^ so mancher Gutachter, ^*^ wieso, ^*^ da ^*^ das Ergebnis ^*^ augenscheinlich ^*^ nicht ^*^ verbessert wurde.

Eine Biologin mit Doktortitel ^*^ hatten ^*^ die Naturschützer ^*^ auf einer Fachkonferenz ^*^ äußerst heftig kritisiert, ^*^ aber ^*^ die anderen Teilnehmer ^*^ erinnerten ^*^ sich ^*^ nicht, ^*^ wieso, ^*^ zumal ^*^ die Diskussion ^*^ offenbar ^*^ abseits des Podiums ^*^ stattfand.

Eine Biologin mit Doktortitel ^*^ hatte ^*^ die Naturschützer ^*^ auf einer Fachkonferenz ^*^ äußerst heftig kritisiert, ^*^ aber ^*^ die anderen Teilnehmer ^*^ erinnerten ^*^ sich ^*^ nicht, ^*^ wieso, ^*^ zumal ^*^ die Diskussion ^*^ offenbar ^*^ abseits des Podiums ^*^ stattfand.

Einen Biologen mit Doktortitel ^*^ hatten ^*^ die Naturschützer ^*^ auf einer Fachkonferenz ^*^ äußerst heftig kritisiert, ^*^ aber ^*^ die anderen Teilnehmer ^*^ erinnerten ^*^ sich ^*^ nicht, ^*^ wieso, ^*^ zumal ^*^ die Diskussion ^*^ offenbar ^*^ abseits des Podiums ^*^ stattfand.

Ein Biologe mit Doktortitel ^*^ hatte ^*^ die Naturschützer ^*^ auf einer Fachkonferenz ^*^ äußerst heftig kritisiert, ^*^ aber ^*^ die anderen Teilnehmer ^*^ erinnerten ^*^ sich ^*^ nicht, ^*^ wieso, ^*^ zumal ^*^ die Diskussion ^*^ offenbar ^*^ abseits des Podiums ^*^ stattfand.

Eine Patientin mit unklaren Symptomen ^*^ hatten ^*^ die Krankenschwestern ^*^ dem behandelnden Arzt zufolge ^*^ mehrfach angeschrien, ^*^ aber ^*^ es ^*^ war ^*^ nicht ^*^ zu ergründen, ^*^ wieso, ^*^ obwohl ^*^ seitdem ^*^ schon ^*^ mehrere Gespräche ^*^ geführt wurden.

Eine Patientin mit unklaren Symptomen ^*^ hatte ^*^ die Krankenschwestern ^*^ dem behandelnden Arzt zufolge ^*^ mehrfach angeschrien, ^*^ aber ^*^ es ^*^ war ^*^ nicht ^*^ zu ergründen, ^*^ wieso, ^*^ obwohl ^*^ seitdem ^*^ schon ^*^ mehrere Gespräche ^*^ geführt wurden.

Einen Patienten mit unklaren Symptomen ^*^ hatten ^*^ die Krankenschwestern ^*^ dem behandelnden Arzt zufolge ^*^ mehrfach angeschrien, ^*^ aber ^*^ es ^*^ war ^*^ nicht ^*^ zu ergründen, ^*^ wieso, ^*^ obwohl ^*^ seitdem ^*^ schon ^*^ mehrere Gespräche ^*^ geführt wurden.

Ein Patient mit unklaren Symptomen ^*^ hatte ^*^ die Krankenschwestern ^*^ dem behandelnden Arzt zufolge ^*^ mehrfach angeschrien, ^*^ aber ^*^ es ^*^ war ^*^ nicht ^*^ zu ergründen, ^*^ wieso, ^*^ obwohl ^*^ seitdem ^*^ schon ^*^ mehrere Gespräche ^*^ geführt wurden.

Eine Teenagerin ohne Schulabschluss ^*^ hatten ^*^ die Talentsucher ^*^ in der Bewerbungsphase ^*^ angeschrieben, ^*^ aber ^*^ der Programmverantwortliche ^*^ fragte ^*^ sich ^*^ ernsthaft, ^*^ wozu, ^*^ denn ^*^ bemerkenswerte Fähigkeiten ^*^ wurden ^*^ an keiner Stelle ^*^ erwähnt.

Eine Teenagerin ohne Schulabschluss ^*^ hatte ^*^ die Talentsucher ^*^ in der Bewerbungsphase ^*^ angeschrieben, ^*^ aber ^*^ der Programmverantwortliche ^*^ fragte ^*^ sich ^*^ ernsthaft, ^*^ wozu, ^*^ denn ^*^ bemerkenswerte Fähigkeiten ^*^ wurden ^*^ an keiner Stelle ^*^ erwähnt.

Einen Teenager ohne Schulabschluss ^*^ hatten ^*^ die Talentsucher ^*^ in der Bewerbungsphase ^*^ angeschrieben, ^*^ aber ^*^ der Programmverantwortliche ^*^ fragte ^*^ sich ^*^ ernsthaft, ^*^ wozu, ^*^ denn ^*^ bemerkenswerte Fähigkeiten ^*^ wurden ^*^ an keiner Stelle ^*^ erwähnt.

Ein Teenager ohne Schulabschluss ^*^ hatte ^*^ die Talentsucher ^*^ in der Bewerbungsphase ^*^ angeschrieben, ^*^ aber ^*^ der Programmverantwortliche ^*^ fragte ^*^ sich ^*^ ernsthaft, ^*^ wozu, ^*^ denn ^*^ bemerkenswerte Fähigkeiten ^*^ wurden ^*^ an keiner Stelle ^*^ erwähnt.

Eine Straßenhündin mit schwarzem Fell ^*^ hatten ^*^ die Kinder ^*^ bis an den Rand des Dorfes ^*^ verfolgt, ^*^ aber ^*^ niemand ^*^ konnte ^*^ sich ^*^ erklären, ^*^ weshalb, ^*^ zumal ^*^ das Tier ^*^ sich ^*^ normalerweise ^*^ vor Menschen versteckte.

Eine Straßenhündin mit schwarzem Fell ^*^ hatte ^*^ die Kinder ^*^ bis an den Rand des Dorfes ^*^ verfolgt, ^*^ aber ^*^ niemand ^*^ konnte ^*^ sich ^*^ erklären, ^*^ weshalb, ^*^ zumal ^*^ das Tier ^*^ sich ^*^ normalerweise ^*^ vor Menschen versteckte.

Einen Straßenhund mit schwarzem Fell ^*^ hatten ^*^ die Kinder ^*^ bis an den Rand des Dorfes ^*^ verfolgt, ^*^ aber ^*^ niemand ^*^ konnte ^*^ sich ^*^ erklären, ^*^ weshalb, ^*^ zumal ^*^ das Tier ^*^ sich ^*^ normalerweise ^*^ vor Menschen versteckte.

Ein Straßenhund mit schwarzem Fell ^*^ hatte ^*^ die Kinder ^*^ bis an den Rand des Dorfes ^*^ verfolgt, ^*^ aber ^*^ niemand ^*^ konnte ^*^ sich ^*^ erklären, ^*^ weshalb, ^*^ zumal ^*^ das Tier ^*^ sich ^*^ normalerweise ^*^ vor Menschen versteckte.

Eine Violinistin des Nationalorchesters ^*^ hatten ^*^ die Konzertbesucher ^*^ während der halbstündigen Pause ^*^ heimlich fotografiert, ^*^ aber ^*^ der Beitrag ^*^ verriet ^*^ leider ^*^ nicht, ^*^ weshalb, ^*^ sondern ^*^ befasste ^*^ sich ^*^ eher ^*^ mit der Bildqualität.

Eine Violinistin des Nationalorchesters ^*^ hatte ^*^ die Konzertbesucher ^*^ während der halbstündigen Pause ^*^ heimlich fotografiert, ^*^ aber ^*^ der Beitrag ^*^ verriet ^*^ leider ^*^ nicht, ^*^ weshalb, ^*^ sondern ^*^ befasste ^*^ sich ^*^ eher ^*^ mit der Bildqualität.

Einen Violinisten des Nationalorchesters ^*^ hatten ^*^ die Konzertbesucher ^*^ während der halbstündigen Pause ^*^ heimlich fotografiert, ^*^ aber ^*^ der Beitrag ^*^ verriet ^*^ leider ^*^ nicht, ^*^ weshalb, ^*^ sondern ^*^ befasste ^*^ sich ^*^ eher ^*^ mit der Bildqualität.

Ein Violinist des Nationalorchesters ^*^ hatte ^*^ die Konzertbesucher ^*^ während der halbstündigen Pause ^*^ heimlich fotografiert, ^*^ aber ^*^ der Beitrag ^*^ verriet ^*^ leider ^*^ nicht, ^*^ weshalb, ^*^ sondern ^*^ befasste ^*^ sich ^*^ eher ^*^ mit der Bildqualität.

Eine Korrespondentin des erfolgreichen Nachrichtensenders ^*^ hatten ^*^ die Kollegen ^*^ vor laufender Kamera ^*^ schlechtgemacht, ^*^ aber ^*^ in einem Gespräch ^*^ konnte ^*^ nicht ^*^ festgestellt werden, ^*^ weshalb, ^*^ sodass ^*^ der Konflikt ^*^ trotz aller Entschuldigungen ^*^ ohne Zweifel ^*^ weiterhin bestehen blieb.

Eine Korrespondentin des erfolgreichen Nachrichtensenders ^*^ hatte ^*^ die Kollegen ^*^ vor laufender Kamera ^*^ schlechtgemacht, ^*^ aber ^*^ in einem Gespräch ^*^ konnte ^*^ nicht ^*^ festgestellt werden, ^*^ weshalb, ^*^ sodass ^*^ der Konflikt ^*^ trotz aller Entschuldigungen ^*^ ohne Zweifel ^*^ weiterhin bestehen blieb.

Einen Korrespondenten des erfolgreichen Nachrichtensenders ^*^ hatten ^*^ die Kollegen ^*^ vor laufender Kamera ^*^ schlechtgemacht, ^*^ aber ^*^ in einem Gespräch ^*^ konnte ^*^ nicht ^*^ festgestellt werden, ^*^ weshalb, ^*^ sodass ^*^ der Konflikt ^*^ trotz aller Entschuldigungen ^*^ ohne Zweifel ^*^ weiterhin bestehen blieb.

Ein Korrespondent des erfolgreichen Nachrichtensenders ^*^ hatte ^*^ die Kollegen ^*^ vor laufender Kamera ^*^ schlechtgemacht, ^*^ aber ^*^ in einem Gespräch ^*^ konnte ^*^ nicht ^*^ festgestellt werden, ^*^ weshalb, ^*^ sodass ^*^ der Konflikt ^*^ trotz aller Entschuldigungen ^*^ ohne Zweifel ^*^ weiterhin bestehen blieb.

Eine Autorin aus Bolivien ^*^ hatten ^*^ die vier Literaturwissenschaftler ^*^ in einem 2500-Seiten-Werk ^*^ zitiert, ^*^ aber ^*^ noch ^*^ kann ^*^ niemand ^*^ sagen, ^*^ wo, ^*^ da ^*^ der Text ^*^ bislang ^*^ seltsamerweise ^*^ verschollen blieb.

Eine Autorin aus Bolivien ^*^ hatte ^*^ die vier Literaturwissenschaftler ^*^ in einem 2500-Seiten-Werk ^*^ zitiert, ^*^ aber ^*^ noch ^*^ kann ^*^ niemand ^*^ sagen, ^*^ wo, ^*^ da ^*^ der Text ^*^ bislang ^*^ seltsamerweise ^*^ verschollen blieb.

Einen Autor aus Bolivien ^*^ hatten ^*^ die vier Literaturwissenschaftler ^*^ in einem 2500-Seiten-Werk ^*^ zitiert, ^*^ aber ^*^ noch ^*^ kann ^*^ niemand ^*^ sagen, ^*^ wo, ^*^ da ^*^ der Text ^*^ bislang ^*^ seltsamerweise ^*^ verschollen blieb.

Ein Autor aus Bolivien ^*^ hatte ^*^ die vier Literaturwissenschaftler ^*^ in einem 2500-Seiten-Werk ^*^ zitiert, ^*^ aber ^*^ noch ^*^ kann ^*^ niemand ^*^ sagen, ^*^ wo, ^*^ da ^*^ der Text ^*^ bislang ^*^ seltsamerweise ^*^ verschollen blieb.

Eine Studentin mit außergewöhnlichen Leistungen ^*^ hatten ^*^ die Professoren ^*^ laut Stellungnahme des Instituts ^*^ tatkräftig unterstützt, ^*^ aber ^*^ es ^*^ war ^*^ nicht ^*^ zu erfahren, ^*^ wobei, ^*^ da ^*^ der Projektverantwortliche ^*^ nicht ^*^ für Nachfragen ^*^ zu erreichen ist.

Eine Studentin mit außergewöhnlichen Leistungen ^*^ hatte ^*^ die Professoren ^*^ laut Stellungnahme des Instituts ^*^ tatkräftig unterstützt, ^*^ aber ^*^ es ^*^ war ^*^ nicht ^*^ zu erfahren, ^*^ wobei, ^*^ da ^*^ der Projektverantwortliche ^*^ nicht ^*^ für Nachfragen ^*^ zu erreichen ist.

Einen Studenten mit außergewöhnlichen Leistungen ^*^ hatten ^*^ die Professoren ^*^ laut Stellungnahme des Instituts ^*^ tatkräftig unterstützt, ^*^ aber ^*^ es ^*^ war ^*^ nicht ^*^ zu erfahren, ^*^ wobei, ^*^ da ^*^ der Projektverantwortliche ^*^ nicht ^*^ für Nachfragen ^*^ zu erreichen ist.

Ein Student mit außergewöhnlichen Leistungen ^*^ hatte ^*^ die Professoren ^*^ laut Stellungnahme des Instituts ^*^ tatkräftig unterstützt, ^*^ aber ^*^ es ^*^ war ^*^ nicht ^*^ zu erfahren, ^*^ wobei, ^*^ da ^*^ der Projektverantwortliche ^*^ nicht ^*^ für Nachfragen ^*^ zu erreichen ist.

Eine Schwimmerin mit zwei Beinprothesen ^*^ hatten ^*^ die Komiteemitglieder ^*^ bezüglich der geplanten Werbekampagne ^*^ kontaktiert, ^*^ aber ^*^ es ^*^ bleibt ^*^ äußerst ^*^ schleierhaft, ^*^ wann, ^*^ zumal ^*^ das Schriftstück ^*^ angeblich ^*^ zwischenzeitlich ^*^ verloren gegangen ist.

Eine Schwimmerin mit zwei Beinprothesen ^*^ hatte ^*^ die Komiteemitglieder ^*^ bezüglich der geplanten Werbekampagne ^*^ kontaktiert, ^*^ aber ^*^ es ^*^ bleibt ^*^ äußerst ^*^ schleierhaft, ^*^ wann, ^*^ zumal ^*^ das Schriftstück ^*^ angeblich ^*^ zwischenzeitlich ^*^ verloren gegangen ist.

Einen Schwimmer mit zwei Beinprothesen ^*^ hatten ^*^ die Komiteemitglieder ^*^ bezüglich der geplanten Werbekampagne ^*^ kontaktiert, ^*^ aber ^*^ es ^*^ bleibt ^*^ äußerst ^*^ schleierhaft, ^*^ wann, ^*^ zumal ^*^ das Schriftstück ^*^ angeblich ^*^ zwischenzeitlich ^*^ verloren gegangen ist.

Ein Schwimmer mit zwei Beinprothesen ^*^ hatte ^*^ die Komiteemitglieder ^*^ bezüglich der geplanten Werbekampagne ^*^ kontaktiert, ^*^ aber ^*^ es ^*^ bleibt ^*^ äußerst ^*^ schleierhaft, ^*^ wann, ^*^ zumal ^*^ das Schriftstück ^*^ angeblich ^*^ zwischenzeitlich ^*^ verloren gegangen ist.

Eine Mathematikerin mit Programmierkenntnissen ^*^ hatten ^*^ die Seitenbetreiber ^*^ über die Sicherheitslücke ^*^ informiert, ^*^ aber ^*^ der Staatsanwalt ^*^ wollte ^*^ genau ^*^ wissen, ^*^ wann, ^*^ da ^*^ dies ^*^ für den Tathergang ^*^ womöglich ^*^ äußerst entscheidend war.

Eine Mathematikerin mit Programmierkenntnissen ^*^ hatte ^*^ die Seitenbetreiber ^*^ über die Sicherheitslücke ^*^ informiert, ^*^ aber ^*^ der Staatsanwalt ^*^ wollte ^*^ genau ^*^ wissen, ^*^ wann, ^*^ da ^*^ dies ^*^ für den Tathergang ^*^ womöglich ^*^ äußerst entscheidend war.

Einen Mathematiker mit Programmierkenntnissen ^*^ hatten ^*^ die Seitenbetreiber ^*^ über die Sicherheitslücke ^*^ informiert, ^*^ aber ^*^ der Staatsanwalt ^*^ wollte ^*^ genau ^*^ wissen, ^*^ wann, ^*^ da ^*^ dies ^*^ für den Tathergang ^*^ womöglich ^*^ äußerst entscheidend war.

Ein Mathematiker mit Programmierkenntnissen ^*^ hatte ^*^ die Seitenbetreiber ^*^ über die Sicherheitslücke ^*^ informiert, ^*^ aber ^*^ der Staatsanwalt ^*^ wollte ^*^ genau ^*^ wissen, ^*^ wann, ^*^ da ^*^ dies ^*^ für den Tathergang ^*^ womöglich ^*^ äußerst entscheidend war.

Eine Abgeordnete der Landtagsfraktion ^*^ hatten ^*^ die Finanzbeamten ^*^ in einem offenen Brief ^*^ gemaßregelt, ^*^ aber ^*^ fünfzig Jahre später ^*^ erscheint ^*^ es ^*^ unverständlich, ^*^ weshalb, ^*^ da ^*^ aus heutiger Sicht ^*^ wohl ^*^ kein Fehlverhalten ^*^ vorlag.

Eine Abgeordnete der Landtagsfraktion ^*^ hatte ^*^ die Finanzbeamten ^*^ in einem offenen Brief ^*^ gemaßregelt, ^*^ aber ^*^ fünfzig Jahre später ^*^ erscheint ^*^ es ^*^ unverständlich, ^*^ weshalb, ^*^ da ^*^ aus heutiger Sicht ^*^ wohl ^*^ kein Fehlverhalten ^*^ vorlag.

Einen Abgeordneten der Landtagsfraktion ^*^ hatten ^*^ die Finanzbeamten ^*^ in einem offenen Brief ^*^ gemaßregelt, ^*^ aber ^*^ fünfzig Jahre später ^*^ erscheint ^*^ es ^*^ unverständlich, ^*^ weshalb, ^*^ da ^*^ aus heutiger Sicht ^*^ wohl ^*^ kein Fehlverhalten ^*^ vorlag.

Ein Abgeordneter der Landtagsfraktion ^*^ hatte ^*^ die Finanzbeamten ^*^ in einem offenen Brief ^*^ gemaßregelt, ^*^ aber ^*^ fünfzig Jahre später ^*^ erscheint ^*^ es ^*^ unverständlich, ^*^ weshalb, ^*^ da ^*^ aus heutiger Sicht ^*^ wohl ^*^ kein Fehlverhalten ^*^ vorlag.

Eine Sanitäterin des Rettungsteams ^*^ hatten ^*^ die Feuerwehrleute ^*^ nachdrücklich ^*^ um Hilfe gebeten, ^*^ aber ^*^ man ^*^ verstand ^*^ später ^*^ nicht, ^*^ warum, ^*^ bis ^*^ schließlich ^*^ Bildmaterial vom Unglücksort ^*^ das Ausmaß der Verwüstung ^*^ verständlich machte.

Eine Sanitäterin des Rettungsteams ^*^ hatte ^*^ die Feuerwehrleute ^*^ nachdrücklich ^*^ um Hilfe gebeten, ^*^ aber ^*^ man ^*^ verstand ^*^ später ^*^ nicht, ^*^ warum, ^*^ bis ^*^ schließlich ^*^ Bildmaterial vom Unglücksort ^*^ das Ausmaß der Verwüstung ^*^ verständlich machte.

Einen Sanitäter des Rettungsteams ^*^ hatten ^*^ die Feuerwehrleute ^*^ nachdrücklich ^*^ um Hilfe gebeten, ^*^ aber ^*^ man ^*^ verstand ^*^ später ^*^ nicht, ^*^ warum, ^*^ bis ^*^ schließlich ^*^ Bildmaterial vom Unglücksort ^*^ das Ausmaß der Verwüstung ^*^ verständlich machte.

Ein Sanitäter des Rettungsteams ^*^ hatte ^*^ die Feuerwehrleute ^*^ nachdrücklich ^*^ um Hilfe gebeten, ^*^ aber ^*^ man ^*^ verstand ^*^ später ^*^ nicht, ^*^ warum, ^*^ bis ^*^ schließlich ^*^ Bildmaterial vom Unglücksort ^*^ das Ausmaß der Verwüstung ^*^ verständlich machte.

Eine Befürworterin der Steuerreform ^*^ hatten ^*^ die Leiter der betroffenen Behörden ^*^ wiederholt ^*^ verbal angegriffen, ^*^ aber ^*^ es ^*^ bleibt ^*^ völlig ^*^ im Dunkeln, ^*^ weshalb, ^*^ da ^*^ das Wortgefecht ^*^ von beiden Seiten ^*^ überaus unsachlich ^*^ geführt wurde.

Eine Befürworterin der Steuerreform ^*^ hatte ^*^ die Leiter der betroffenen Behörden ^*^ wiederholt ^*^ verbal angegriffen, ^*^ aber ^*^ es ^*^ bleibt ^*^ völlig ^*^ im Dunkeln, ^*^ weshalb, ^*^ da ^*^ das Wortgefecht ^*^ von beiden Seiten ^*^ überaus unsachlich ^*^ geführt wurde.

Einen Befürworter der Steuerreform ^*^ hatten ^*^ die Leiter der betroffenen Behörden ^*^ wiederholt ^*^ verbal angegriffen, ^*^ aber ^*^ es ^*^ bleibt ^*^ völlig ^*^ im Dunkeln, ^*^ weshalb, ^*^ da ^*^ das Wortgefecht ^*^ von beiden Seiten ^*^ überaus unsachlich ^*^ geführt wurde.

Ein Befürworter der Steuerreform ^*^ hatte ^*^ die Leiter der betroffenen Behörden ^*^ wiederholt ^*^ verbal angegriffen, ^*^ aber ^*^ es ^*^ bleibt ^*^ völlig ^*^ im Dunkeln, ^*^ weshalb, ^*^ da ^*^ das Wortgefecht ^*^ von beiden Seiten ^*^ überaus unsachlich ^*^ geführt wurde.

Eine Gegnerin des umstrittenen Staudammprojekts ^*^ hatten ^*^ die Planer ^*^ schließlich ^*^ doch noch überzeugt, ^*^ aber ^*^ es ^*^ herrscht ^*^ Stillschweigen ^*^ darüber, ^*^ wie, ^*^ weil ^*^ niemand ^*^ sich ^*^ dem Verdacht der Bestechlichkeit ^*^ aussetzen will.

Eine Gegnerin des umstrittenen Staudammprojekts ^*^ hatte ^*^ die Planer ^*^ schließlich ^*^ doch noch überzeugt, ^*^ aber ^*^ es ^*^ herrscht ^*^ Stillschweigen ^*^ darüber, ^*^ wie, ^*^ weil ^*^ niemand ^*^ sich ^*^ dem Verdacht der Bestechlichkeit ^*^ aussetzen will.

Einen Gegner des umstrittenen Staudammprojekts ^*^ hatten ^*^ die Planer ^*^ schließlich ^*^ doch noch überzeugt, ^*^ aber ^*^ es ^*^ herrscht ^*^ Stillschweigen ^*^ darüber, ^*^ wie, ^*^ weil ^*^ niemand ^*^ sich ^*^ dem Verdacht der Bestechlichkeit ^*^ aussetzen will.

Ein Gegner des umstrittenen Staudammprojekts ^*^ hatte ^*^ die Planer ^*^ schließlich ^*^ doch noch überzeugt, ^*^ aber ^*^ es ^*^ herrscht ^*^ Stillschweigen ^*^ darüber, ^*^ wie, ^*^ weil ^*^ niemand ^*^ sich ^*^ dem Verdacht der Bestechlichkeit ^*^ aussetzen will.

Eine Soldatin der gegnerischen Streitkräfte ^*^ hatten ^*^ die ausgesandten Kundschafter ^*^ offenbar ^*^ in die Irre geführt, ^*^ aber ^*^ der Befehlshaber ^*^ begriff ^*^ einfach ^*^ nicht, ^*^ wie, ^*^ obwohl ^*^ ihm ^*^ die Finte ^*^ mehrmals ^*^ erklärt worden war.

Eine Soldatin der gegnerischen Streitkräfte ^*^ hatte ^*^ die ausgesandten Kundschafter ^*^ offenbar ^*^ in die Irre geführt, ^*^ aber ^*^ der Befehlshaber ^*^ begriff ^*^ einfach ^*^ nicht, ^*^ wie, ^*^ obwohl ^*^ ihm ^*^ die Finte ^*^ mehrmals ^*^ erklärt worden war. Einen Soldaten der gegnerischen Streitkräfte ^*^ hatten ^*^ die ausgesandten Kundschafter ^*^ offenbar ^*^ in die Irre geführt, ^*^ aber ^*^ der Befehlshaber ^*^ begriff ^*^ einfach ^*^ nicht, ^*^ wie, ^*^ obwohl ^*^ ihm ^*^ die Finte ^*^ mehrmals ^*^ erklärt worden war.

Ein Soldat der gegnerischen Streitkräfte ^*^ hatte ^*^ die ausgesandten Kundschafter ^*^ offenbar ^*^ in die Irre geführt, ^*^ aber ^*^ der Befehlshaber ^*^ begriff ^*^ einfach ^*^ nicht, ^*^ wie, ^*^ obwohl ^*^ ihm ^*^ die Finte ^*^ mehrmals ^*^ erklärt worden war.
